# Clinical neurophysiological interrogation of motor slowing: A critical step towards tuning adaptive deep brain stimulation

**DOI:** 10.1016/j.clinph.2023.04.013

**Published:** 2023-05-16

**Authors:** Laura Alva, Elena Bernasconi, Flavie Torrecillos, Petra Fischer, Alberto Averna, Manuel Bange, Abteen Mostofi, Alek Pogosyan, Keyoumars Ashkan, Muthuraman Muthuraman, Sergiu Groppa, Erlick A. Pereira, Huiling Tan, Gerd Tinkhauser

**Affiliations:** a Department of Neurology, Bern University Hospital and University of Bern, Bern, Switzerland; b MRC Brain Network Dynamics Unit, University of Oxford, Oxford, United Kingdom; c Nuffield Department of Clinical Neurosciences, University of Oxford, Oxford, United Kingdom; d School of Physiology, Pharmacology & Neuroscience, University of Bristol, University Walk, BS8 1TD Bristol, United Kingdom; e Movement Disorders and Neurostimulation, Department of Neurology, University Medical Center of the Johannes Gutenberg University Mainz, 55131 Mainz, Germany; f Neurosciences Research Centre, Molecular and Clinical Sciences Research Institute, St George’s, University of London, London SW17 0RE, United Kingdom; g Department of Neurosurgery, King’s College Hospital, King’s College London, SE59RS, United Kingdom

**Keywords:** Adaptive deep brain stimulation, Closed-loop DBS, Local field potentials, Basal Ganglia, Parkinson’s disease, DBS programming

## Abstract

**Objective:**

Subthalamic nucleus (STN) beta activity (13–30 Hz) is the most accepted biomarker for adaptive deep brain stimulation (aDBS) for Parkinson’s disease (PD). We hypothesize that different frequencies within the beta range may exhibit distinct temporal dynamics and, as a consequence, different relationships to motor slowing and adaptive stimulation patterns. We aim to highlight the need for an objective method to determine the aDBS feedback signal.

**Methods:**

STN LFPs were recorded in 15 PD patients at rest and while performing a cued motor task. The impact of beta bursts on motor performance was assessed for different beta candidate frequencies: the individual frequency strongest associated with motor slowing, the individual beta peak frequency, the frequency most modulated by movement execution, as well as the entire-, low- and high beta band. How these candidate frequencies differed in their bursting dynamics and theoretical aDBS stimulation patterns was further investigated.

**Results:**

The individual motor slowing frequency often differs from the individual beta peak or beta-related movement-modulation frequency. Minimal deviations from a selected target frequency as feedback signal for aDBS leads to a substantial drop in the burst overlapping and in the alignment of the theoretical onset of stimulation triggers (to ~ 75% for 1 Hz, to ~ 40% for 3 Hz deviation).

**Conclusions:**

Clinical-temporal dynamics within the beta frequency range are highly diverse and deviating from a reference biomarker frequency can result in altered adaptive stimulation patterns.

**Significance:**

A clinical-neurophysiological interrogation could be helpful to determine the patient-specific feedback signal for aDBS.

## 1 Introduction

Adaptive deep brain stimulation (aDBS) represents a next generation precision medicine therapy to optimize symptom control in patients with movement and neuropsychiatric disorders (di Biase et al., 2021, Meidahl et al., 2017). This technology should enable the titration of the stimulation according to the temporal manifestation of subcortical or cortical electrophysiological biomarkers (Tinkhauser and Moraud, 2021). Although first experimental clinical trials showed promising results, several questions still need to be resolved until aDBS becomes sufficiently robust and user-friendly for clinical use (Tinkhauser, 2022). One crucial aspect is the best strategy on how to select a patient-specific feedback signal to drive aDBS. Basal ganglia beta activity (13–30 Hz) is the most investigated and established electrophysiological symptom biomarker in Parkinson’s disease (PD) (Brown et al., 2001). Over recent years, a large body of group-level data linked increased beta activity to motor symptoms such as bradykinesia and rigidity (Duchet et al., 2021, Khawaldeh et al., 2022, Kuhn et al., 2006, Neumann et al., 2016, Tinkhauser et al., 2020). In fact, most of the aDBS pilot trials so far capitalized on beta activity as biomarker for the control algorithm (Arlotti et al., 2018, Bocci et al., 2021, Little et al., 2013, Little et al., 2016b, Pina-Fuentes et al., 2017). However, beta activity spans a wide spectral frequency range and the selection of the frequency for the feedback signal has often been arbitrarily either based on the individual beta peak (±a variable range, 2–7 Hz) obtained at rest or the entire beta band (Arlotti et al., 2018, Bocci et al., 2021, Herz et al., 2018, Little et al., 2016a, Little et al., 2013, Petrucci et al., 2020, Pina-Fuentes et al., 2017, Rosa et al., 2017, Velisar et al., 2019). Yet beta activity is often divided into two sub-bands (low beta 13–20 Hz and high beta 21–30 Hz) and preferential functional and anatomical roles such as dopaminergic responsiveness, cortical-subcortical connectivity as well as limb-specificities have been attributed (Fogelson et al., 2006, Oswal et al., 2016, Oswal et al., 2021, Priori et al., 2004, Tinkhauser et al., 2019, Tinkhauser et al., 2018b). Regarding motor symptom specificity, the existing data have not provided clear directions as to whether the low or high beta band is more linked to motor slowing and hence more suitable as biomarker (Neumann et al., 2016, Sure et al., 2021). In addition to the spectral range, also the intrinsic temporal dynamics of beta oscillations, such as the bursting characteristics must be considered. This is particularly motivated by the link between beta bursts and motor performance (Khawaldeh et al., 2022, Lofredi et al., 2022, Tinkhauser et al., 2020, Torrecillos et al., 2018), as well as the positive clinical outcome when stimulation specifically targets these transient neurophysiological states (Little et al., 2016a, Little et al., 2013, Tinkhauser et al., 2017a).

The goal of this work is to support the translation of a patient-tailored stimulation therapy. We hypothesize that different spectral activities within the beta range may exhibit a frequency-dependent relationship to motor slowing and distinct temporal dynamic leading to different aDBS stimulation patterns. To demonstrate this, we contrasted for each subject three individualized frequencies (frequency most associated with motor slowing, beta peak frequency, frequency with the most pronounced movement-related desynchronization) as well as the low-, high- and entire beta band. We assessed the frequency-specific relationship to motor slowing in a motor task and the temporal dynamics were evaluated using the burst overlapping metric and the theoretical aDBS trigger timings. With this work, we aim to create awareness and showcase that a clinical-neurophysiological interrogation may be a critical step to select the individual feedback signal for aDBS.

## 2 Methods

### 2.1 Subjects and surgery

We studied a cohort of 15 patients with PD who underwent bilateral STN-DBS surgery and temporary lead externalization. All subjects have been previously reported (Tinkhauser et al., 2020). Their clinical details are summarized in Supplementary Table 1. Subjects were recruited at three different sites, St. Georges Hospital London (UK), Kings College Hospital London (UK) and Mainz University Hospital (DE). The study was approved by the local ethics committees (Mainz University Hospital: 837.208.17 (11042); UK centres: IRAS 46576) and all subjects gave their written informed consent. The DBS lead implantation approach was center-specific: imaging-guided alone (St. Georges Hospital and Kings College Hospital) or imaging-guided and use of intraoperative electrophysiology (Mainz University Hospital). The implanted DBS leads were either the directional leads from Boston Scientific with three segmented contacts on levels 2 and 3 or the Medtronic 3389 DBS lead (Medtronic Neurological Division) with four platinum-iridium cylindrical surfaces. DBS leads were temporarily externalised for 3–6 days.

### 2.2 Experimental set-up and offline behavioural analysis

**Fig. 1:**
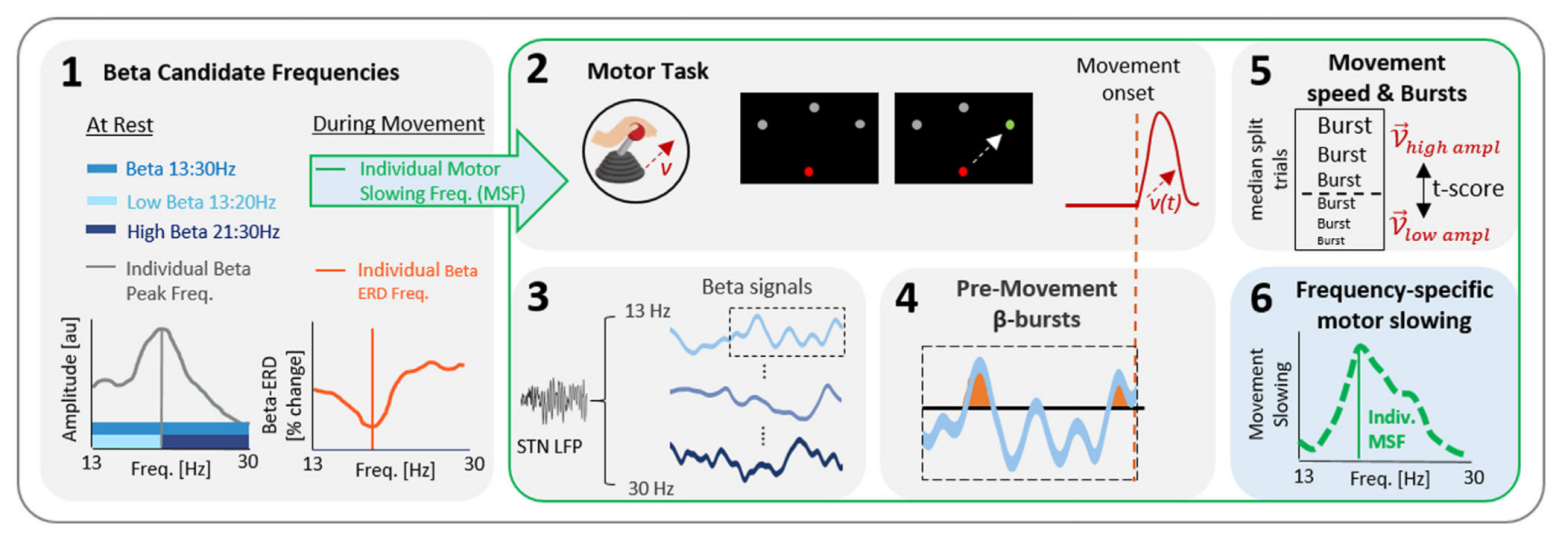
Method: Beta frequency-specific impact on motor slowing. (1) Illustrates the different beta candidate frequencies determined after withdrawal of dopaminergic medication either on the power spectral density at rest (entire beta: 13–30 Hz, low beta: 13–20 Hz, high beta: 21–30 Hz, individual beta peak frequency) or during task-related movements (beta ERD frequency, individual motor slowing frequency). The following steps (2–6) show how the individual motor slowing frequency was determined: (2) Subjects performed multiple trials of a visually-cued joystick task with the instruction to move as fast as possible toward a randomly occurring green cue. The peak velocity of the movement was measured in every trial. (3) STN LFPs were recorded in parallel and further decomposed into the 1 Hz resolution frequency components of the beta frequency range (13–30 Hz). (4) For every trial and beta frequency, beta bursts were derived using the 75th percentile amplitude threshold. For all the bursts occurring in the pre-movement period (2.5 s prior to movement onset until movement onset), the mean burst amplitude was calculated. For trials in which no beta burst occurred, the burst amplitude was set to zero. (5) Separately for every beta frequency, the trials were median split into two groups based on their mean burst amplitude. The movement peak velocity of the two groups of trials were compared and the corresponding t-score (derived from an independent two-sampled t-test) determined, serving as an indicator of frequency-specific motor slowing. (6) The green dashed line illustrates the t-score at each single frequency. The single beta frequency with the strongest association with motor slowing, corresponding to the highest t-score, was termed individual motor slowing frequency (individual MSF). MSF = motor slowing frequency; STN = subthalamic nucleus; aDBS = adaptive Deep Brain Stimulation; ERD = event-related desynchronization.

In this work, we re-used data from a previous study (Tinkhauser et al., 2020) that comprised a visually cued joystick task with different task conditions. The visual cue that triggered the joystick movement either appeared in response to an occurring STN beta burst in real-time (of a pre-defined frequency) or randomly without any specific temporal relationship to beta bursts (Tinkhauser et al., 2020). For the present study, we only used the spectral and behavioural data from the latter condition, in which movements were triggered randomly. This is in line with our scope of investigating the temporal dynamics of beta bursts and the associated motor slowing across different beta frequencies, not restricted to a particular frequency. The details of the experimental set-up and data acquisition and processing are outlined in the original publication (Tinkhauser et al., 2020) and in the Supplementary Material of the present work. All assessments were performed OFF dopaminergic medication. Brain and motion signals were recorded using a TMSi-Porti amplifier (TMS International, Netherlands), sampled at 2048 Hz and common average referenced. LFPs were recorded in a bipolar contact arrangement between the four electrode levels. The bipolar channel further used for the task and analyses was the one with the highest resting beta activity. This step was motivated by evidence linking maximal beta band activity (Shah et al., 2023, Tinkhauser et al., 2018a, Tinkhauser et al., 2019) to the motor region of the STN. During the task, subjects sat in front of a computer monitor with their hand, contralateral to the trigger STN channel, holding a joystick. They were instructed to move the joystick as fast as possible in the direction of a green target (GO-cue) as soon as it appeared on the monitor (Fig. 1, box 2). After initial familiarization with the task, we aimed to obtain a minimum number of 60 trials. Motor performance was assessed by the peak velocity of the joystick movement, defined as the maximum velocity in the direction of the target after movement onset. The median reaction time across subjects was 0.59 s (see Supplementary Fig. 1).

### 2.3 Brain signal processing

LFPs were resampled to 200 Hz and decomposed into their frequency components with a frequency resolution of 1 Hz using a Wavelet transformation (ft_specest_wavelet script in Fieldtrip - Morlet Wavelet, width = 10, gwidth = 5; Donders Institute for Brain, Cognition and Behaviour, 2010). To investigate the frequency-specific temporal dynamics of STN beta activity, we determined the beta burst profile using the previously established threshold-based method (Tinkhauser et al., 2017a, Tinkhauser et al., 2017b, Tinkhauser et al., 2018b, Tinkhauser et al., 2020, Torrecillos et al., 2018). This approach also helps to link the results to threshold-based aDBS algorithms (Little et al., 2016a, Little et al., 2013, Velisar et al., 2019). In this work, we focused on a set of different beta candidate frequencies including the entire- (13–30 Hz), low- (13–20 Hz) and high- (21–30 Hz) beta band, as well as the individual beta peak frequency, the individual frequency with the strongest movement-related beta desynchronization and the individual frequency with the strongest relationship to motor slowing (Fig. 1, box 1). For the investigated beta bands, the corresponding frequency bins have been averaged before the beta burst profile was determined. For the individual beta peak frequency, the beta peak was defined as the local power maximum within the beta frequency range (13–30 Hz). The suggested peaks were visually re-evaluated and only adjusted if they did not correspond to the most evident discrete peak, which was the case in 2 out of 15 subjects. To determine the frequency with the strongest beta modulation related to movement, we calculated for each trial the percentage power change between the movement period (ranging from the movement onset to 200 ms after the movement onset) and the common baseline power (averaged across trials) at −3 to −1 seconds before movement onset. The beta frequency with the highest percent event-related desynchronization (%-beta ERD) was defined as the individual beta ERD frequency. The approach we applied to the individual motor slowing frequency is explained in the section below. Note, the default burst threshold was set as the 75th percentile amplitude of the selected signal. Certain analyses were repeated with the 50th amplitude threshold, which is then specified in the text. The threshold was calculated separately for the rest recording and the trial data, the details on how the latter has been calculated is specified below. Moreover, to avoid the categorization of spontaneous amplitude fluctuations due to noise as beta burst, we excluded bursts shorter than a minimum duration. This was defined as period equal to the duration of 2 oscillatory cycles of the frequency considered (e.g. 100 ms for the 20 Hz bin). For the beta bands (entire, low, high), the minimum burst duration (2 oscillatory cycles) was defined based on the average frequency value of the corresponding beta band (i.e. entire (13–30 Hz) = 21. 5 Hz, low (13–20 Hz) = 16.5 Hz, high (21–30 Hz) = 25.5 Hz).

### 2.4 Beta bursts across frequencies and their impact on motor performance

**Fig. 2:**
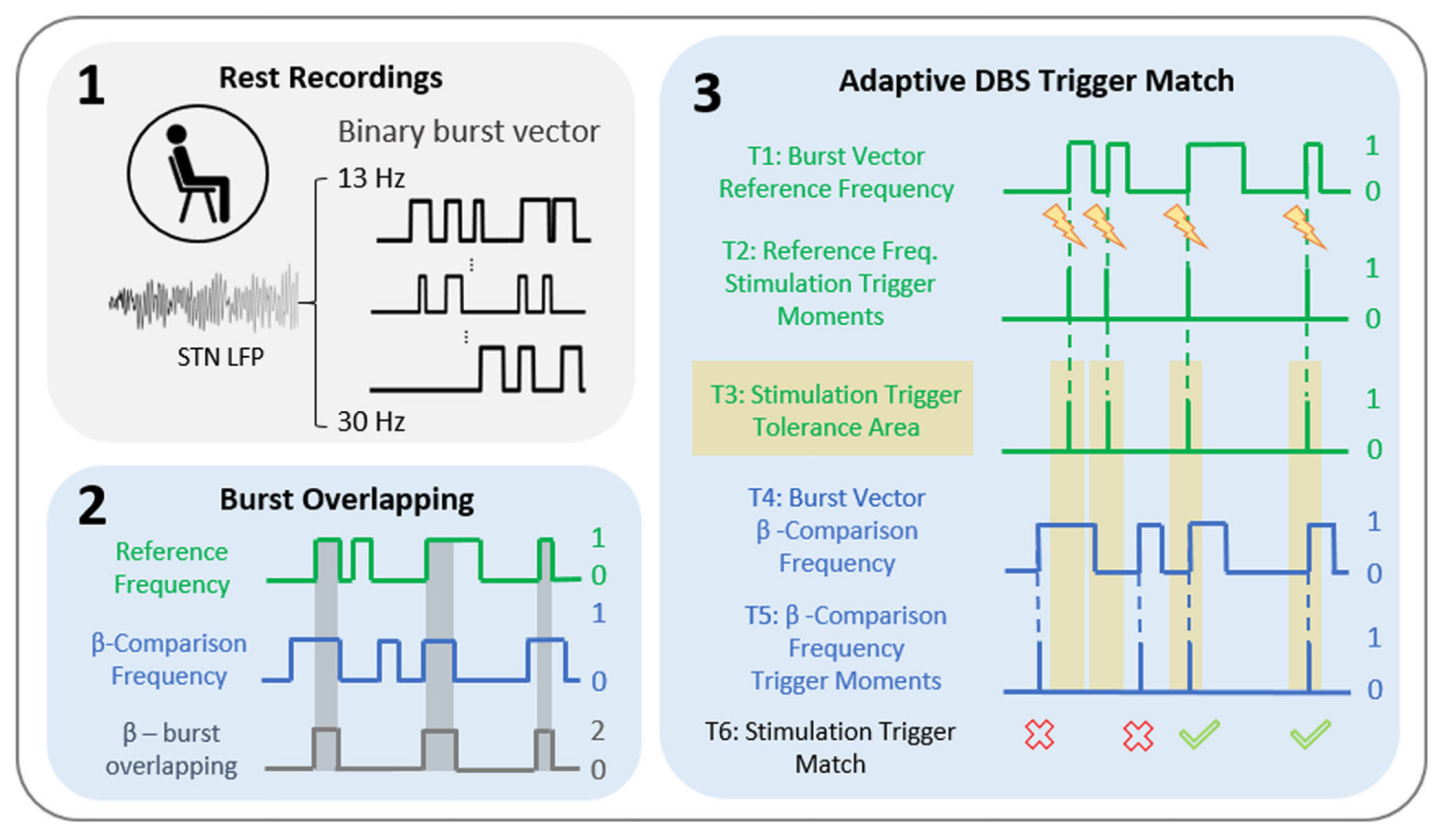
Method: Temporal dynamics across beta frequencies. Schematic illustrating the pipeline to quantify the % burst overlapping and % aDBS Trigger Match. (1) LFPs recorded at rest were decomposed in its beta frequency components ranging from 13-30 Hz. Beta bursts were defined using the 75th percentile amplitude threshold and the signal envelope was transformed into a binary (no burst = 0, burst = 1) burst vector. (2) Illustrates how to derive the % burst overlapping metric (%OVL): The temporal similarities between two beta candidate frequencies (i.e. low-, high-, entire beta band, individual MSF, individual beta peak and beta ERD frequency) either selected as reference or beta comparison frequency, were quantified as %OVL. (3) Illustration of the method to derive the % aDBS Trigger Match between the theoretical adaptive stimulation triggers (stimulation triggered once burst is detected) resulting from the reference frequency and the triggering of the beta comparison frequencies. T1: binary burst vector of the reference frequency. T2: marks the theoretical aDBS trigger timings of the reference frequency. T3: shows an arbitrary “Stimulation Trigger Tolerance Area” (trigger timing ± 100 ms) defined as trigger period that would still provide clinically beneficial stimulation. T4: shows the binary burst vector of the beta comparison frequency. T5: shows the corresponding aDBS trigger timings of the beta comparison frequencies. T6: shows the “Stimulation Trigger Match” i.e. the alignment of the aDBS trigger of the beta comparison frequency with the “Stimulation Trigger Tolerance Area”. MSF = motor slowing frequency; aDBS = adaptive Deep Brain Stimulation; ERD = event-related desynchronization; T = Trace.

A first step of this study was to identify the individual frequency of the beta range (13 to 30 Hz) that was most strongly associated with movement slowing. The methodological pipeline for this is illustrated in Fig. 1 (box 2 to 6). For all beta frequencies and bands of each single trial, we determined the mean amplitude and the duration of the bursts that occurred in the 2.5 s prior to the movement onset. We chose this period as the cumulative occurrence of beta bursts in this epoch was previously linked with movement velocity (Tinkhauser et al., 2020). Note, the beta burst related motor slowing was also derived for a range of shorter burst detection time windows and the resulting movement speed modulation curves were compared for consistency to each other. The 75th percentile threshold was calculated based on the LFP amplitude of the pre-movement period (-3 s to −1 s prior to movement) averaged across trials, separately for each subject and frequency. We opted to use this common threshold over the trial-based threshold to allow for a better comparability of beta bursts across trials. To calculate the relationship between the peak velocity of the joystick movements and the pre-movement beta burst properties, we applied the following method: For each beta frequency and subject, trials were median split into two groups based on the mean burst amplitude. For trials without detected beta burst, the burst amplitude was set to zero. The peak velocities of these two groups were compared using an independent two-samples t-test and the resulting t-score of this test was then used as proxy of the frequency-specific slowing, meaning a higher positive t-score indicates that a higher mean burst amplitude preceded stronger slowing. Following this, we defined the frequency with the strongest association to motor slowing (i.e. the frequency with the highest t-score value) as “individual motor slowing frequency (individual MSF)”. While we opted for the frequency-specific relationship to motor slowing using the t-score on the median-split trials in line with the original publication (Tinkhauser et al., 2020), the correlative trend between peak velocity and burst amplitude (or movement-related ERD) quantified as Spearman’s correlation coefficients could have been used as alternative method (see Supplementary Material). Additionally, the entire analysis pipeline was repeated using burst duration instead of the mean burst amplitude to determine the relationship between peak velocity and spectral frequency, which is also presented in the Supplementary Material. The next methodological steps after having defined the different beta candidate frequencies is to calculate how they differ in their temporal dynamics and alignment in their theoretical adaptive stimulation trigger timings (aDBS Trigger Match) (Fig. 2).

### 2.5 Temporal dynamics across beta frequencies

To investigate the temporal relationship of the different beta frequencies, we applied the beta burst overlapping metric on the signals at rest (Tinkhauser et al., 2018b). The percentage of overlapping bursts was calculated pairwise between all different beta candidate frequencies, as illustrated in the processing pipeline (Fig. 2). To this end, we transformed the signals into a binary burst vector. Hereby, we attributed the value “1” to burst periods and the value “0” to non-burst periods. The binary burst vectors of two beta candidate frequencies were added together, with the resulting value “2” for periods with burst overlapping. The percentage of overlapping bursts (%OVL) was then defined as the percentage duration of overlapping bursts relative to the cumulative burst duration of the reference frequency. The %OVL was calculated in both directions, i.e. with both of the two beta candidate frequencies once as the reference, and the two %OVL values were averaged. Secondly, we determined the change in %OVL with incremental 1 Hz step deviations from the individual beta candidate frequencies (MSF, beta peak and beta ERD). We also contrasted the effective %OVL with the overlapping by chance calculated according to a previously established method (Tinkhauser et al., 2018b) by setting a random break point in the binary burst vector and reversing and joining the two segments together. With this method, we maintained the integrity of the bursts (i.e. number and duration of bursts), while their temporal occurrence became random. We then calculated the %OVL by chance for the different beta frequency-bins (13–30 Hz) and repeated this procedure for 1000 iterations. The final value of %OVL due to chance was then calculated by taking the mean across all frequencies and iterations. To further test whether the frequency resolution of the signal decomposition impacts upon the %OVL, we contrasted the results of the resting state signals decomposed using different wavelet widths (5,10,15).

### 2.6 Adaptive DBS trigger match

The stimulation trigger timings during aDBS depend on the stimulation algorithm used and the temporal dynamics of the selected feedback signal. Similar as for the %OVL, the percentage aDBS trigger match (%trigger match) was calculated between all different beta candidate frequencies as illustrated in Fig. 2. To do this, we considered the starting points of the bursts at the reference frequency as the optimal aDBS stimulation trigger time points. In addition, we considered a stimulation trigger area around the trigger time point of ± 100 ms. This area reflects an arbitrary timespan of tolerance for triggering stimulation for which no pre-existing approximative data exist. Therefore, we empirically chose a duration corresponding to two beta cycles (of a 20 Hz burst) on both sides of the trigger moment. We then assessed how often the burst onset of one candidate frequency coincided with the stimulation trigger area of the reference frequency and reported the result as %trigger match. This was calculated in both directions, i.e. using both of the two beta frequencies once as the reference, and the two %trigger match values were averaged. An additional analysis comprised the change in %trigger match with incremental 1 Hz step deviations from the individual beta candidate frequencies (MSF, beta peak, beta ERD). We also considered the change in %trigger match when the same reference signals got expanded using an incremental number of neighbouring frequencies (±1 to 7 Hz). Similar as above we contrasted the effective %trigger match with the trigger match by chance. Exemplary analyses were repeated for beta bursts defined by the 50th percentile amplitude burst thresholds.

### 2.7 Comparisons and statistical analysis

All statistical analyses were performed using MATLAB (version R2020b; MathWorks). To test the relationship between frequency-specific indices of motor slowing (t-score values, see section 2.4) and power-spectral density (PSD) as well as movement-related power modulation (beta ERD), we applied a Spearman correlation. To evaluate the group level statistical significance of the t-score across the whole beta frequency range (13–30 Hz), we used a cluster-based permutation procedure. To this end, t-scores were compared against zero and p-values were derived by randomly permuting the sign of the t-scores for a subset of subjects 5000 times. For each frequency point, the t-statistic of the actual mean difference was computed based on the distribution of the 5000 differences resulting from permutation. The resulting p-values were then corrected for multiple comparison by determining the supra-threshold clusters (pre-cluster threshold: p < 0.05) for each permutation, and then storing the sum of the t-statistics within these clusters to form a distribution of the largest supra-threshold-cluster values. Finally, the 95th percentile of this distribution served as statistical threshold for the map of the actual t-statistics of the real difference (Maris and Oostenveld, 2007). Thus, only those significant clusters that exceeded the threshold survived the cluster-based permutation correction. Furthermore, to test whether the t-score values of selected frequencies significantly differ from chance at the single subject level, we also calculated permutation statistics. For this comparison, we randomly shuffled trial indices and recalculated the t-scores derived by the t-test, which was repeated for 5000 iterations. P-values of the individual t-scores were then calculated based on how many of the permutation-distribution t-scores were larger than the original t-score (Ernst, 2004). To evaluate the changes in %OVL and %trigger match when deviating from individual beta candidate frequencies, we applied a Friedman test followed by pair-wise Wilcoxon signed rank tests for between group comparison. For multiple comparisons, FDR (false discovery rate) corrected p-values were reported.

## 3 Results

### 3.1 Frequency dependent motor performance across the beta range

**Fig. 3:**
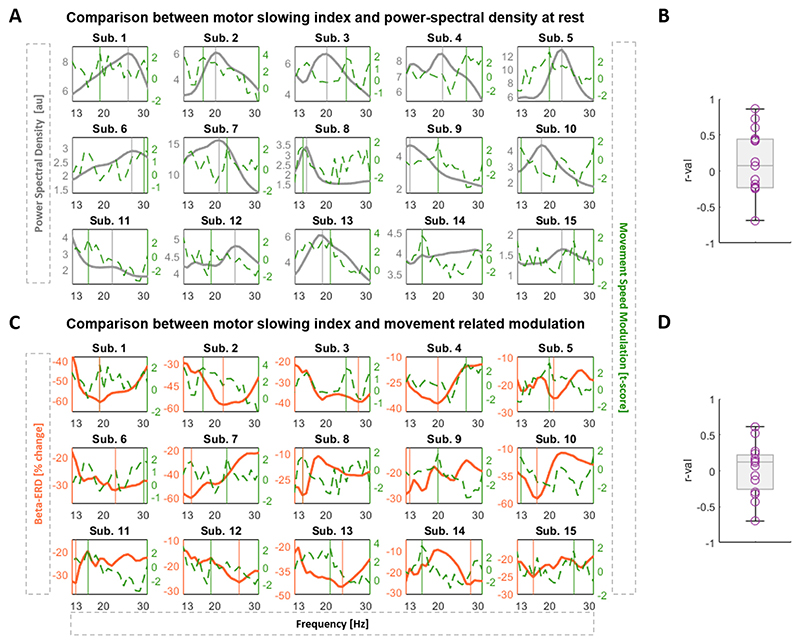
Clinical and spectral profile across subjects. **A)** Illustrates for every subject (n = 15) and beta frequency the PSD of the LFPs recorded at rest (grey line) and the burst amplitude dependent motor modulation represented as t-score (dashed green line). A high t-score indicates a stronger association between burst amplitude and motor slowing. In some instances (e.g. sub. 8), both the PSD and t-score curves show a similar magnitude across the beta range, while in others (e.g. sub. 10) even an inverse relationship can be seen. The vertical lines mark the individual beta peak frequency (grey line) and the individual MSF i.e. frequency strongest associated with the motor slowing (green line). **B)** Shows the distribution of the r-values derived from the Spearman’s correlation between the individual PSD curve and t-score curves across subjects. The median r-value is 0.07, ranging from −0.69 to 0.86. **C)** Illustrates for every subject and beta frequency the averaged movement-related beta desynchronization, the % beta ERD (orange line), and the burst amplitude dependent motor modulation represented as t-score (dashed green line, same as above). The vertical lines mark the individual frequency with the strongest beta ERD (orange line) and the individual MSF (green line). **D)** Shows the distribution of the r-values derived for each subject from the Spearman’s correlation between the individual beta ERD curve and t-score curves. The median r-value is 0.12, ranging from −0.70 to 0.61. Note for B) and D): If the curves would consistently co-fluctuate, i.e. if the peaks and troughs would approximately co-occur, we should only see positive r-values that are close to 1 for B, and only negative r-values, closer to −1 for D (because in the orange line the trough denotes the beta frequency that would be selected based on movement-related ERD). The fact that the r-values are both positive and negative, ranging a broad span, summarizes that the peaks (and ERD troughs), and thus the selected aDBS frequency, can be very different. Note, bursts are defined using the 75th percentile amplitude threshold. Sub. = Subjects; AU = arbitrary unit; LFP = local field potentials; PSD = power spectral density; ERD = event-related desynchronization.

**Fig. 4:**
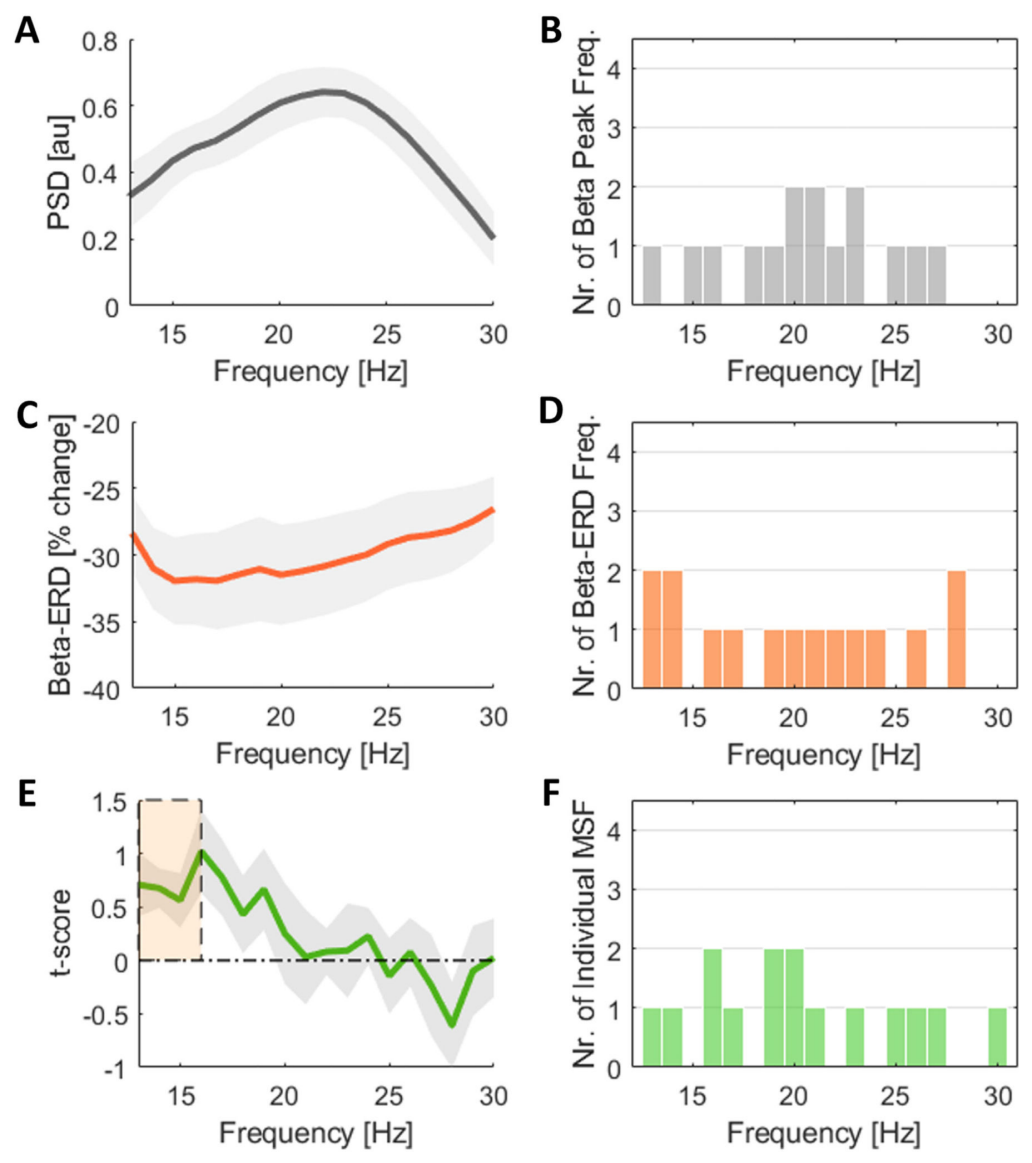
Beta activity spectral characteristics and impact on motor slowing across beta frequencies. **A)** Shows the resting state PSD (13–30 Hz) averaged across patients (n = 15), with a beta peak at 22 Hz. Values are represented as mean ± SEM. **B)** Illustrates the distribution of the visually defined individual beta peaks. Seven of 15 subjects have the beta peak in the low beta band (13–20 Hz) and 8 of 15 subjects have the beta peak in the high beta band (21–30 Hz) **C)** Shows the movement related beta modulation (%-beta ERD) averaged across subjects (n = 15), with the strongest ERD present at 15 Hz (-31.95% ± 3.19%). Values are represented as mean ± SEM. **D)** Illustrates the distribution of the individual maximal ERD frequencies across the beta range. In 8 out of 15 subject the strongest ERD is falling in the low beta band and in 7 out of 15 subjects it is falling in the high beta band. **E)** Shows the t-scores as indicator of motor slowing across the beta frequency range and averaged across subjects (n = 15). Values are represented as mean ± SEM. The strongest spectral association with motor slowing at the group level is found in the lower beta frequency range, with a significant cluster ranging from 13 to 16 Hz. **F)** Illustrates the distribution of the individual MSFs, i.e. the individual beta frequency strongest associated with motor slowing. In 9 of 15 subjects the individual MSF is in the low beta range and in 6 out of 15 cases in the high beta range. In 4 out of 15 subjects it is located in the significant cluster area. PSD = power spectral density; ERD = event-related desynchronization; SEM = standard error of the mean.

In this study, we investigated the burst dynamics of the different beta frequencies and their impact on motor performance. This we believe may be a critical aspect for the selection of the optimal feedback signal for aDBS. We determined the t-score value as indicator of the beta-burst dependent motor slowing (indexed by positive values) for all frequencies within the beta range and related this to the corresponding PSD at rest and movement-related ERD. In Fig. 3A, we contrast the individual PSD curves with the t-score based movement speed modulation curves. This shows a variable relationship, with some subjects having an overall alignment between PSD and movement speed modulation curves (e.g. subject 8), while for other subjects the two curves appear unrelated (e.g. subject 10). This lack of a consistent trend is further supported by the wide distribution of the correlation coefficients (median r-val = 0.07, ranging from −0.69 to 0.86) when correlating the PSD and the t-score curves within subjects (Fig. 3B). When comparing movement-related modulation curves with the movement speed modulation curves, the correlation shows a similar large variation (Fig. 3C-D). We made similar observations when using burst duration instead of burst amplitude to determine the frequency-specific slowing (Supplementary Fig. 2). Note, the spectral-behavioural relationship (dashed green line) illustrated in Fig. 3A/C are represented as t-scores, however expressing this relationship using the correlation coefficients would provide similar results (Supplementary Fig. 3). Moreover, we contrasted the single-subject and group level observations. The highest beta power at rest is found at 22 Hz for the group average (Fig. 4A), while on a single-subject level, the beta peaks are distributed across the entire beta frequency range, with 7/15 located in the low beta band and 8/15 in the high beta band (Fig. 4B). Similar for the movement-related power modulation, the strongest ERD in the group average is located at 15 Hz, while 8 of the 15 subjects show the strongest ERD frequency in the low beta band and 7 in the high beta band (Fig. 4C-D).

### 3.2 Akinesia-specific beta frequency

**Fig. 5:**
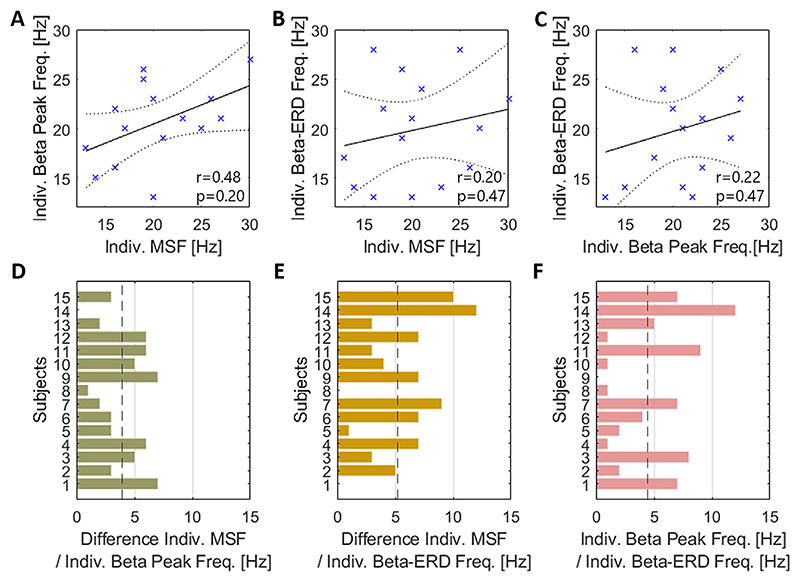
Spectral relationship between individual MSF, beta peak and beta ERD frequencies. **A)** Spearman’s correlation between the individual MSFs and the individual beta peak frequencies (r = 0.48, p = 0.20). **B)** Spearman’s correlation between the individual MSF and the frequencies with strongest beta ERD (r = 0.20, p = 0.47). **C)** Spearman’s correlation between the individual beta peak frequencies and the frequencies with strongest beta ERD (r = 0.22, p = 0.47). **D)** Illustrates for every subject the absolute difference between the individual MSF and the individual beta peak frequency, which on average corresponds to 3.9 Hz (dashed line). **E)** Shows the absolute difference between the individual MSF and the individual beta ERD frequency, which is 5.2 Hz on average. **F)** Shows the absolute difference between the individual beta peak frequency and the individual beta ERD frequency, that on average corresponds to 4.5 Hz. MSF = motor slowing frequency; ERD = event-related desynchronization; SEM = standard error of the mean.

In principle, we could assume that the optimal feedback signal selected for aDBS should be maximally indicative for the target symptom state. While the spectral-behavioural link between the beta band and akinetic-rigid symptoms in PD has been repeatedly shown, the question remains whether there is an individual preferred target frequency within the beta range that would help to increase the temporal precision of aDBS. Following this concept, we applied a simple joystick motor task to determine the beta frequency which is most associated with motor slowing by relating the mean burst amplitude with the peak velocity of movements, indexed as t-score (Fig. 1). The frequency associated with the strongest motor slowing (i.e. highest t-score) we termed as “individual motor slowing frequency” (individual MSF). This is conceptually like the selection process of the patient-specific stimulation settings. On the group average, the t-score curves show the strongest association with motor slowing in the lower beta frequency range, with a significant cluster ranging from 13-16 Hz (Fig. 4E). This frequency varies from subject to subject and is distributed across the beta spectral range, falling into the low beta range in 9 and into the high beta range in 6 out of 15 subjects (Fig. 4F). In fact, only 4 out of 15 subjects had their individual MSF located in the significant group-level cluster (Fig. 4E). Note, the movement speed modulation curves were also comparable when different burst detection windows were applied (Supplementary Table 2). Moreover, we saw a positive, but not significant trend when comparing the individual MSF with the individual beta peak frequencies detected at rest (r-val = 0.48, p-val = 0.20) (Fig. 5A). No clear trend was found between the individual MSF and the beta ERD frequencies (r-val = 0.20, p-val = 0.47) (Fig. 5B). On average, the absolute difference between the individual MSF and the individual beta peaks is 3.9 Hz with one overlap, and 5.2 Hz between the individual MSF and the beta ERD frequencies with 2 overlaps (Fig. 5D-E). Note, subject 4 and 5 are a subset of patients with a tremor-dominant form of PD, who in contrast to the akinetic-rigid type PD patients, might require biomarkers other than beta activity to index the tremor (Hirschmann et al., 2017, Shah et al., 2018). However, with regard to motor slowing, the difference between their individual MSF and the beta peak frequency is 6 Hz and 4 Hz, between their individual MSF and the beta ERD frequency 7 Hz and 1 Hz, which is within the range of the remaining cohort. Interestingly, the relationship between the individual beta peak and the beta ERD frequencies is not consistent within subjects (r = 0.22, p = 0.47), yet the average deviation is 4.5 Hz with one overlap (Fig. 5C, F).

Note, the same pipeline has been replicated for using the mean burst duration instead of mean burst amplitude to determine the individual MSF, which provided similar results (Supplementary Figs. 4, 5).

**Fig. 6:**
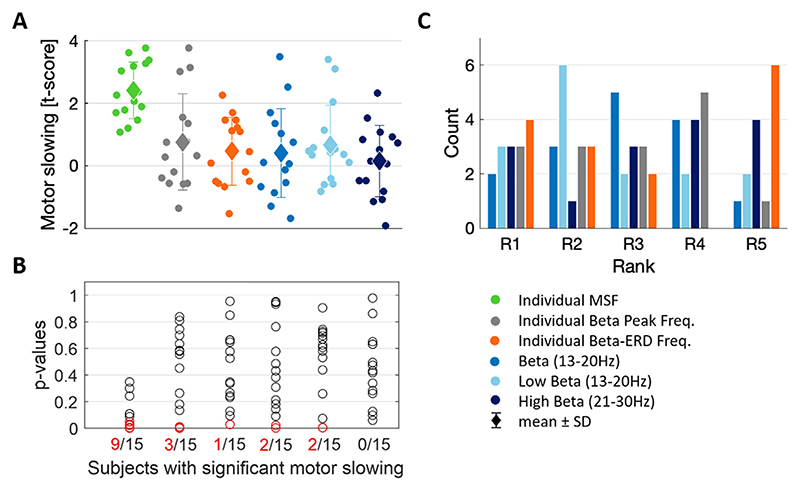
Motor slowing related to the beta candidate frequencies. **A)** Illustrates the distribution of the t-score based motor slowing for the different beta candidate frequencies (individual MSF, individual beta peak, individual beta ERD, as well as the entire (13–30 Hz), low (13–20 Hz) and high (21–30 Hz) beta band) across subjects (n = 15). The mean t-score value ± SD is 2.41 ± 0.90 for the individual MSF, 0.75 ± 1.54 for the individual peak frequency, 0.46 ± 1.10 for the individual beta ERD frequency, 0.41 ± 1.43 for the entire beta band, 0.67 ± 1.28 for the low beta band, and 0.15 ± 1.14 for the high beta band. **B)** Quantifies the number of subjects in whom the frequency-specific t-scores are significantly different from a within-subject permutation distribution, separately for all of the beta candidate frequencies. Nine of 15 subjects show a significant slowing for the individual MSF, 3/15 for the beta peak frequency at rest, 1/15 for the beta ERD frequency, 2/15 for the entire beta band, 2/15 for the low beta band, and 0/15 for the high beta band (red circles mark the significant p-values < 0.05). **C)** Shows the rank distribution of the frequency-specific motor slowing for the 5 beta comparison frequencies when ranking their t-scores within each subject. Rank 1 (R1) corresponds to the highest t-score (strongest association to motor slowing) while R5 represents the candidate frequency with the weakest association to motor slowing. MSF = motor slowing frequency; ERD = event-related desynchronization.

Next, we compared the distribution of t-scores of the different beta candidate frequencies (Fig. 6A). The mean t-score for the individual MSF outperforms the other beta comparison frequencies by design. To further investigate how robust this frequency-dependent motor slowing is, we tested whether the frequency-specific t-scores of the different beta candidate frequencies are significantly different when tested against a surrogate distribution at the single subject level (Fig. 6B). This is important because random variability in our data might result in positive t-scores, which might not necessarily reflect a true, reproducible difference in movement speed based on preceding beta activity. To test how likely the t-scores reflect a reproducible difference, we took all velocity values and randomly permuted them across trials, resulting in a distribution of t-scores of which half are positive and negative for each subject. Only if the original t-score would be at the very outer edge of this distribution (≤5% of the permutation t-scores are more extreme than the original one), it would be considered significant. This revealed a significant p-value in 9 out of 15 subjects for the individual MSF (also including the two tremor-dominant patients), while the number of subjects with significant frequency-specific t-score values were 3 for the individual beta peak and 1 for the individual beta ERD frequency, 2 for the entire beta, 2 for the low, and 0 for the high beta band. Finally, Fig. 6C shows the rank distribution of the motor slowing for the 5 beta comparison frequencies after ranking their t-scores within each subject (rank 1 = strongest, rank 5 = lowest association to motor slowing). In cases in which the individual beta peak and individual beta ERD frequency correspond to the same frequency bin, the same rank was attributed. The individual MSF has been excluded from this analysis, as it would show the highest rank by design. Descriptively, we observe a slight tendency of low beta being skewed towards better ranks, while the beta ERD often appears lowest ranked. The remaining candidate frequencies are rather similarly distributed across the ranks.

### 3.3 Beta envelope dynamics

**Fig. 7:**
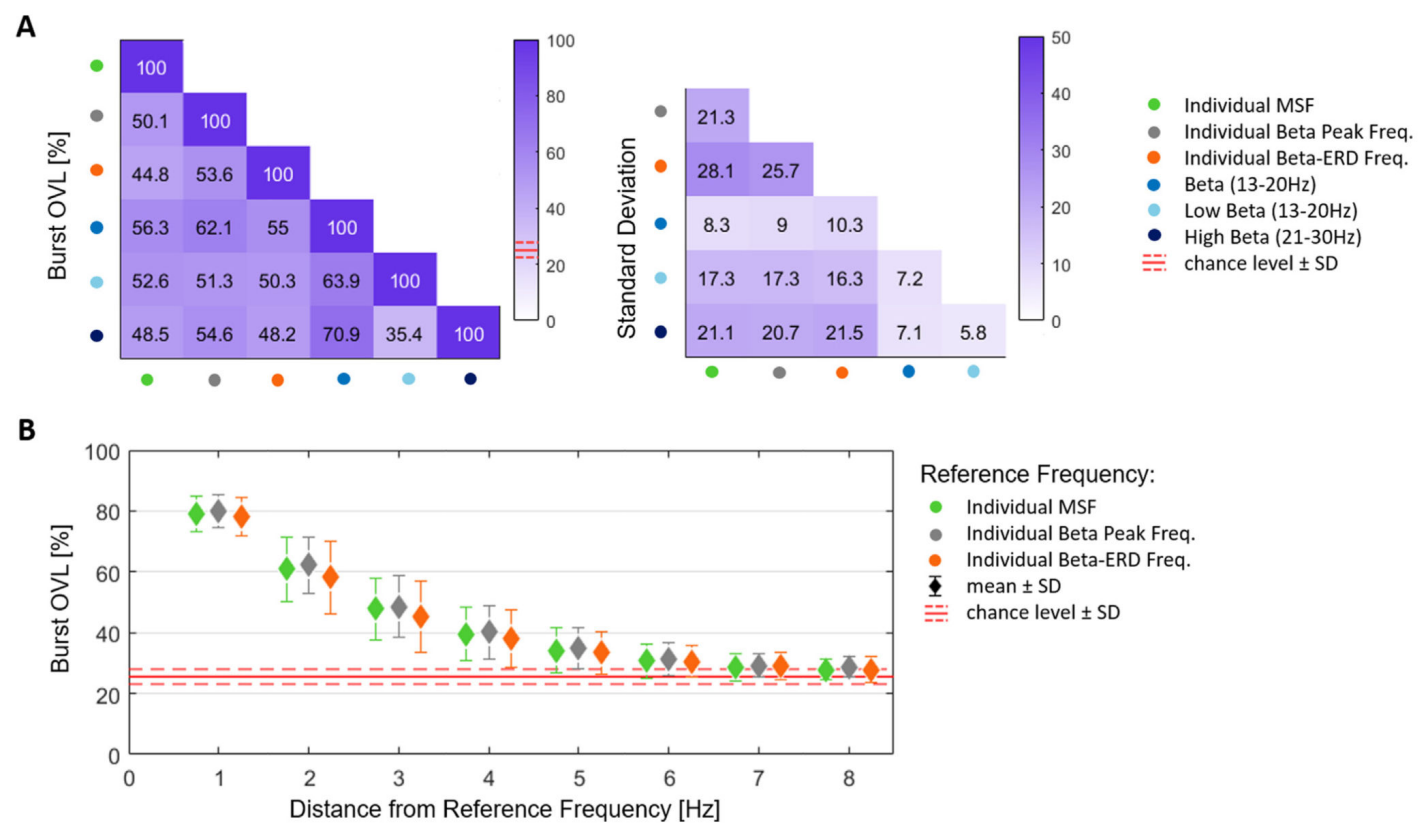
Burst Overlapping. **A)** The matrix (left) illustrates the average % burst overlapping (%OVL) between the different beta candidate frequencies (MSF, beta peak, beta ERD, entire beta (13–30 Hz), low beta (13–20 Hz) and high beta band (21–30 Hz)), averaged across subjects (n = 15). Note that a 100% OVL corresponds to identical beta burst dynamic of both beta candidate frequencies. The chance level ± 1 SD corresponded to 25.54% ± 2.50%. The %OVL ranges from 35.4% (between low beta and high beta) to 70.9% (below entire beta – high beta). The matrix (right) illustrates the corresponding standard deviation values. **B)** Illustrates the frequency-bin wise change in mean %OVL (±SD) with incremental deviation from the individual reference frequencies: the individual MSF (green), the individual beta peak frequency (grey), and the individual beta ERD frequency (orange). Note, the % changes derived from the deviations to both the left and right side have been averaged. The %OVL by chance ± 1 SD is depicted by the horizontal red lines. Friedman’s test revealed a significant decrease in %OVL when deviating from the individual beta candidate frequency (X = 255.29; df = 7; p < 0.001), without significant differences in the group-comparisons. Values are represented as mean ± SD. Bursts are defined using the 75th percentile amplitude threshold. MSF = motor slowing frequency; ERD = event-related desynchronization; OVL = overlapping; SD = standard deviation.

Next, we set out to understand the temporal dynamics of the different beta candidate frequencies. To elaborate on this, we calculated the percentage burst overlapping (%OVL) between the beta candidate frequencies across each other (Fig. 7A). A “100% matched bursting dynamic” (i.e. 100% burst overlap) would result from identical temporal dynamics of two candidate frequencies. The overall level by chance corresponds to 25.54% (SD: ±2.50%). The highest %OVL was found between the entire beta and the high beta band (70.9%) and the lowest %OVL between the high beta and low beta band (35.4%). We further investigated the bin-wise change in %OVL with incremental deviation from the individual beta candidate frequencies (MSF, beta peak, beta ERD) when used as reference signal (Fig. 7B). We found that a deviation of 1 Hz from the individual MSF leads to a drop in %OVL of 79.15%, which further decreases to 47.86% when deviating 3 Hz. This same trend was seen when the beta peak frequency was used as reference, with a reduction to 80.10% and 48.60% after 1 Hz and 3 Hz deviation respectively. Similar, for the beta ERD as reference signal, the reduction of %OVL was 78.17% after 1 Hz and 45.26% after 3 Hz deviation. A Friedman’s test was carried out to evaluate the decrease in OVL when deviating from the individual beta candidate frequencies (X = 255.29; df = 7; p < 0.001). A within-group comparison between different beta candidate frequencies revealed no significant differences after correcting for multiple comparisons (Supplementary Table 3). Note, the % change derived from the deviations to both the left and right side of the individual MSF have been averaged since the difference between both sides was small (see Supplementary Fig. 6). As the frequency bin-wise decay in % OVL could further depend on the spectral resolution of the frequency decomposition, we replicated the same analyses using a set of different wavelet parameters. This revealed a steeper %OVL slope for higher frequency resolution and a flattening of the slope for lower frequency resolution (Supplementary Fig. 7).

### 3.4 Frequency-dependent aDBS stimulation triggering

**Fig. 8:**
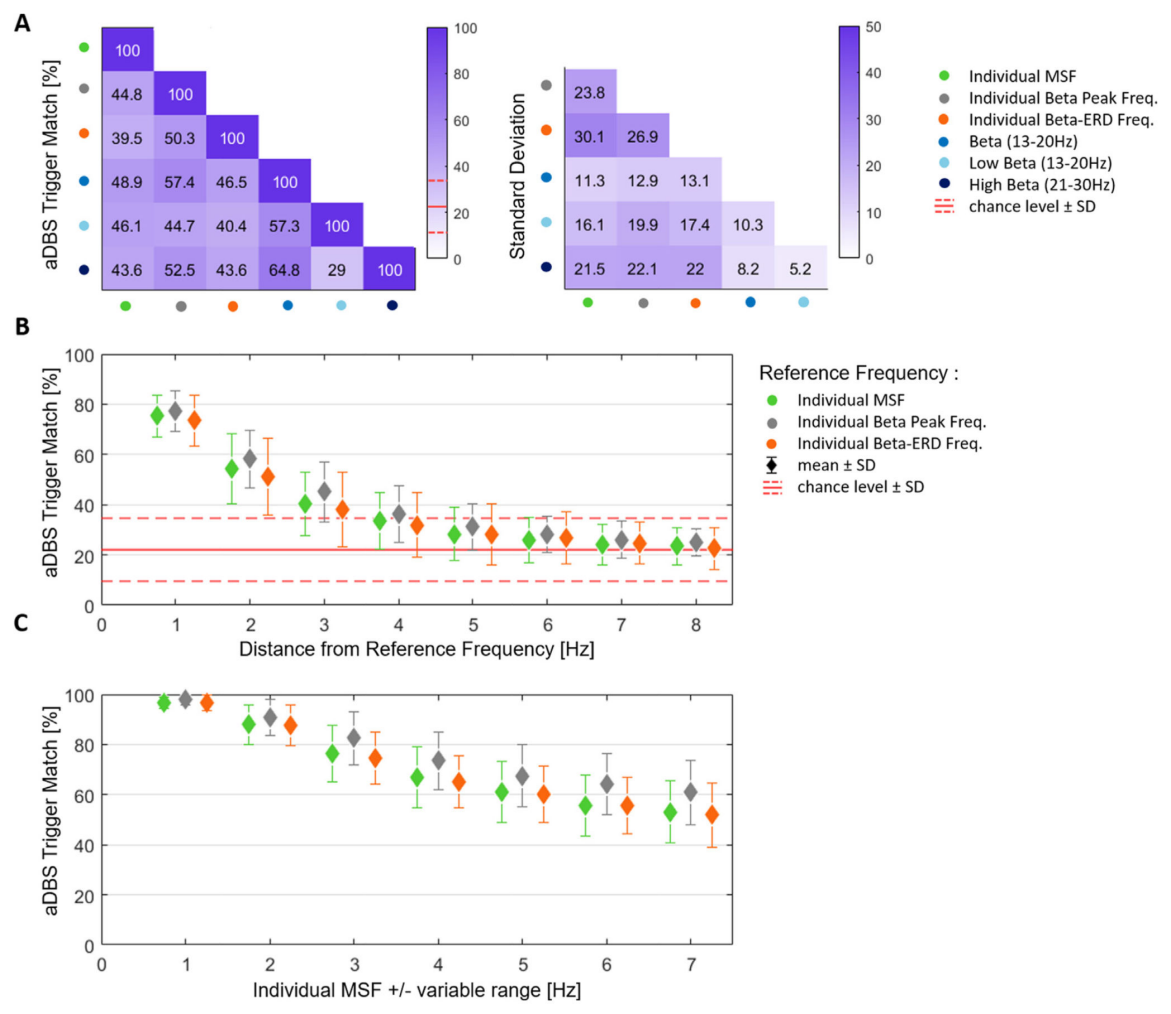
Adaptive DBS Trigger Match. **A)** The matrix (left) illustrates the average % aDBS trigger match between the different beta candidate frequencies (MSF, beta peak, beta ERD, entire beta (13–30 Hz), low beta (13–20 Hz) and high beta band (21–30 Hz)), averaged across subjects (n = 15). The %trigger match is defined by how often the burst onset (stimulation trigger) of one candidate frequency coincides with the stimulation trigger tolerance area of the comparison frequency (and vice versa). The chance level ± 1 SD corresponded to 22.07% ± 12.63%. The %trigger match ranges from 29% (low beta – high beta) to 64.8% (entire beta – high beta). The matrix (right) illustrates the corresponding standard deviation values. **B)** Illustrates the frequency-bin wise change in mean % aDBS trigger match (±SD) with incremental deviation from the individual reference frequencies: the individual MSF (green), the individual beta peak frequency (grey), and the individual beta ERD frequency (orange). Note, the % changes derived from the deviations to both the left and right side have been averaged. The horizontal red lines depict the %trigger match by chance ± 1 SD. Friedman’s test revealed a significant decrease in %trigger match when deviating from the individual beta candidate frequency (X = 226.11; df = 7; p < 0.001), while the post hoc comparison showed no significant difference between the groups. **C)** Illustrates the mean %change (±SD) in trigger-match comparing the single individual reference frequencies (MSF, beta peak, beta ERD) with a same reference signal but combined with an incremental number of neighbouring frequency bins (±1 to 7 Hz). Friedman’s test revealed a significant decrease in % trigger match when combining the target frequency with an incremental number of neighbouring frequency bins (X = 223.6; df = 6; p < 0.001). The post hoc comparison showed a significant difference between the beta peak and beta ERD group at position 1, 3, 4, 5, 6 and 7 Hz. Values are represented as mean ± SD. Bursts are defined using the 75th percentile amplitude threshold. MSF = motor slowing frequency; ERD = event-related desynchronization; SD = standard deviation.

Here we propose that the selection of the feedback signal might be critical for the optimal outcome of aDBS since the precise timing of the stimulation trigger depends on the temporal bursting dynamics. This is of particular importance for “fast control” algorithms, such as threshold-based control policies (Little et al., 2013, Velisar et al., 2019). To demonstrate the impact of the beta burst dynamics on aDBS trigger timings, we computed the %trigger match between all possible pairs of beta candidate frequencies (Fig. 2). In contrast to the burst overlapping metric, this approach is intentionally focused on the critical starting period of the burst, because once stimulation is triggered, the further evolution of the burst (considered in the burst OVL) is likely modulated by the stimulation itself (Tinkhauser et al., 2017a). The overall chance level was 22.07% (SD: ±12.63%). Of all the combinations of the beta candidate frequencies, the highest %trigger match was found between the entire beta and the high beta band (64.77%) and the lowest %trigger match between the low beta and high beta band (28.99%) (Fig. 8A). Similar as for the %OVL, we further investigated the frequency bin-wise change in %trigger match with incremental deviation from the individual beta candidate frequencies (MSF, beta peak, beta ERD) used as reference signals (Fig. 8B). We found that a deviation of 1 Hz from the individual MSF leads to a drop in %trigger match to 75.34%, which further decreases to 40.29% when deviating 3 Hz. This same trend was seen when the beta peak frequency was used as reference instead, with a reduction to 77.26% and 45.08 % after 1 Hz and 3 Hz deviation respectively. Similar, for the beta ERD as reference signal, the reduction of %trigger match was 73.51% after 1 Hz and 37.88% after 3 Hz deviation. A Friedman’s test was performed to evaluate the decrease in trigger match when deviating from the individual beta candidate frequency (X = 226.11; df = 7; p < 0.001). A within-group comparison between different beta frequency candidates revealed no significant differences after correcting for multiple comparisons (Supplementary Table 4). Note, the mean trigger match falls below chance level (±1 SD) after deviation of 4 Hz from the individual MSF and beta ERD frequency and at 5 Hz deviation from the individual beta peak. To increase the comparability of these results to previous aDBS clinical trials in which the 50th percentile amplitude threshold was applied (Little et al., 2016a, Little et al., 2013, Pina-Fuentes et al., 2017), we repeated the analyses for that threshold level as well (Supplementary Fig. 8). Finally, as mentioned above, the selection of the aDBS feedback signal frequency does often include a variable range of neighbouring frequencies. We therefore computed the %change in trigger match comparing the single individualized frequencies with a set of frequencies entailing an incremental number of neighbouring frequency bins (Fig. 8C). For the individual MSF as single reference frequency, the %trigger match decrease to 96.62% with 1 Hz bin, to 76.17% with 3 Hz bins and to 52.98% with 7 Hz bins. With the individual beta peak as single reference frequency, the %trigger match decrease to 97.80% with 1 Hz bin, to 82.46% with 3 Hz bins and to 60.92% with 7 Hz bins. Lastly, when using the individual beta ERD as reference signal, the %trigger match decreases to 96.51% with 1 Hz bin, to 74.63% with 3 Hz bins and to 51.82% with 7 Hz bins. A Friedman’s test was conducted to evaluate the decrease in trigger match when comparing the single individualized frequencies with a set of frequencies entailing an incremental number of neighbouring frequency bins (X = 223.6; df = 6; p < 0.001). A within-group comparison between different beta frequency candidates and after correcting for multiple comparisons, revealed significant differences between the beta peak and beta ERD for the %trigger match difference at the individual frequency ± the following range of neighbouring frequencies: 1, 3, 4, 5, 6 and 7 Hz (Supplementary Table 5).

## 4 Discussion

This work highlights the potential utility of a patient-specific and data-driven feedback signal selection approach to translate clinical aDBS regimes. We present several findings that suggest a one-size-fits-all beta activity biomarker for aDBS might not be optimal in all cases. First, we confirm the individual resting beta peak frequency and the frequency with the strongest movement related modulation can be highly variable across subjects. Secondly, the beta frequency that was strongest associated with motor slowing could not simply be predicted from these spectral properties. Third, different frequencies within the beta range may exhibit distinct temporal dynamics with a frequency-dependent relationship to motor slowing. Fourth, variations in temporal dynamics across beta frequencies also lead to substantial variations in aDBS trigger timings. Consequently, deviating from a reference biomarker frequency can result in altered adaptive stimulation patterns that potentially lead to less beneficial stimulation. We believe that aDBS could benefit from a standardized clinical-neurophysiological interrogation step to determine the individually most beneficial feedback signal as part of the aDBS programming procedure and that studies specifically testing this hypothesis are warranted.

### 4.1 Frequency-specificity of clinical effects

Beta bursts have been shown to impact motor performance and were associated with slowing and movement decrements in PD (He et al., 2020, Lofredi et al., 2019, Tinkhauser et al., 2020, Torrecillos et al., 2018, Yeh et al., 2020). The present results further highlight that spectro-behavioural associations depend on the exact beta frequency or range of frequencies used to derive beta bursts, as this gives rise to different temporal dynamics relative to movements. Group-level data demonstrating functional segregation within the beta band revealed a stronger modulation to the dopaminergic tone for the low beta range (13 to 20 Hz) (Priori et al., 2004), while the high beta range (21 to 30 Hz) is more involved in cortical-subcortical coupling (Fogelson et al., 2006, Oswal et al., 2016, Tinkhauser et al., 2018b). With regard to motor impairment, the functional distinction is less clear, as studies either linked increased amplitude in the low (Neumann et al., 2016) or the high beta band (Sure et al., 2021) to reduced motor performance. This functional segregation is also supported by the diverse temporal dynamics between low and high beta, with only 35.4% of concordance seen in the current study. With regard to motor slowing, our group-level analyses showed a significant relationship between motor slowing and burst amplitude for the frequency range spanning from 13-16 Hz, which overlaps with results described in (Neumann et al., 2016). However, on a subject level, the frequency strongest associated with motor slowing coincided with this significant group-level cluster in only 4 out of 15 cases, while for the remaining cases it spread across the entire beta band. Moreover, although previous data demonstrated that both the beta peak frequency as well as the beta ERD frequency are associated with motor slowing (Devos et al., 2006, Doyle et al., 2005, Shah et al., 2023, Tinkhauser et al., 2020, Torrecillos et al., 2018), these spectral properties may not necessarily index the frequency with the individual strongest association to motor slowing. Thus, to determine the patient- and symptom-specific frequency (or range), a dedicated assessment might be required.

### 4.2 Temporal dynamics of beta activity and impact upon adaptive DBS

It has previously been observed that temporal matching of the stimulation periods with the occurrence of beta bursts is critical to interfere with pathological burst synchronisation and to obtain a clinical improvement, while intermittent but randomly delivered stimulation is not clinically beneficial (Little et al., 2013, Tinkhauser et al., 2017a). In many of the previous aDBS pilot trials, the individual beta peak ± an arbitrary range (from 2 to 7 Hz) or the entire beta band were chosen as feedback signal. The present work shows that there is a substantial mismatch between the trigger time points between the different beta candidate frequencies. Moreover, regardless of which frequency would be chosen as target feedback signal (individual motor slowing frequency, beta peak or beta ERD frequency), there is relevant increase in stimulation trigger error when deviating from this target frequency, that reaches the chance level after 4 Hz of deviation. Thus, the configuration of aDBS without prior knowledge of the symptom-specificity of the frequency range might result in suboptimal clinical efficacy. In fact, the clinical improvement reported in previous aDBS studies (Little et al., 2016a, Little et al., 2013, Velisar et al., 2019) may not necessarily be the best that could have been achieved, assuming potential stimulation trigger errors as the ground truth feedback signal frequency was not objectively assessed. However, selecting the symptom-specific frequency is only one procedural step toward the configuration of the feedback signal. Further parameters such as the frequency resolution of the signal decomposition, the smoothing of the feedback signal, as well as the width of the frequency range (target frequency ± arbitrary number of neighbouring frequencies) all impact upon the feedback signal properties. For example, lowering the frequency resolution by selecting a wider frequency range as feedback signal could potentially be advantageous in case minor frequency shifts occur during stimulation itself and levodopa ON/OFF transitions (Iskhakova et al., 2021). In summary, troubleshooting strategies for clinically non-beneficial aDBS should include neurophysiological testing to identify the optimal beta frequency to avoid ineffective stimulation regimes.

### 4.3 Clinical-neurophysiological interrogation

It has to be emphasized that deploying aDBS regimes is complex and as opposed to conventional DBS, it requires many additional procedural steps (Tinkhauser, 2022). We can expect that in the coming years, the setup of aDBS will require many more hours per patient than currently necessary for conventional DBS. The selection of the feedback signal to drive the aDBS algorithm is one of them. While different symptom biomarkers could be embedded in aDBS control policies (Ding et al., 2022, Swann et al., 2018, Tinkhauser and Moraud, 2021, Wiest et al., 2020, Wiest et al., 2021), the use of beta activity is currently closest to clinical implementation. However, as illustrated here, the temporal dynamics of beta bursting and the clinical-spectral link varies across the beta frequency range and the selection of the feedback frequency solely based on the power spectrum at rest or beta ERD may potentially not suffice. To avoid a time-consuming trial-and-error approach for choosing the individual symptom-specific biomarkers, a standardized data-driven method may quicken this process. A comprehensive neurophysiological setup for PD patients may consider LFP recordings at rest, the modulation of the spectrum OFF/ON levodopa as aDBS control algorithms need to be capable of handling both states and its transitions (Duchet et al., 2021, Lofredi et al., 2018, Tinkhauser et al., 2017b), OFF/ON stimulation (Feldmann et al., 2022, Muthuraman et al., 2020, Tinkhauser et al., 2017a, Wiest et al., 2020), motion (Shah et al., 2023), as well as neuropsychiatric assessments (Ricciardi et al., 2021). In addition, we propose that a dedicated task could serve as interrogative procedure to assess the spectral-clinical link qualitatively and quantitatively. Such an approach also needs to consider different assessments to account for the varying clinical phenomenologies of patients (Tinkhauser and Moraud, 2021). Tremor-dominant PD patients likely require a different set of feedback signals than akinetic-rigid type PD patients (Hirschmann et al., 2017, Shah et al., 2018). In addition to any short-term in-hospital assessment, future devices will provide the option of long-term neurophysiological and behavioural measurements executed in the ambulatory setting. This will allow clinicians to better quantify and contextualize oscillatory biomarkers during medication and circadian changes as well as various physical activities (Gilron et al., 2021a, Gilron et al., 2021b, Tinkhauser and Moraud, 2021, van Rheede et al., 2022). Moreover, patients’ clinical and spectral profiles are highly heterogeneous and a single biomarker such as beta activity will unlikely be able to comprehensively address all symptoms. Hence, machine learning-based optimization strategies may additionally be required in the future (He et al., 2021, Merk et al., 2022, Shah et al., 2023, Tinkhauser and Moraud, 2021). Despite recent technological advances, a sustainable and user-friendly method to set-up and recalibrate aDBS can only evolve in synergy between clinicians, scientists, engineers and industry partners and much more work is needed to implement the technology in routine clinical care.

## 5 Limitations

The present work represents a framework toward a standardized clinical-neurophysiological interrogation to program aDBS. We would like to reemphasize that the design of such an assessment has yet to be established, validated and translated in one simple and accessible clinical tool. Note, it remains speculative whether the individual motor slowing frequency, as presented here, is indeed superior when used as feedback signal for aDBS. Thus, the validation also needs to include a comprehensive evaluation of the clinical efficacy of aDBS using different frequencies as control signal. The task used in the present study entailed relatively small-distance and visually cued joystick rather than spontaneous movements. Repetitive uncued movements with a larger range of motion might be better suited in identifying a bradykinesia-related frequency since PD patients generally are more impaired in self-initiated movements than externally triggered ones (Currà et al., 1997). It also remains to be tested to which extent the movement speed related beta peak frequencies differ from the resting beta peak when patients are in the ON medication state. Differences in movement speed based on the individual motor slowing frequency were only significant in 9/15 subjects, but not for the remaining subjects. This may be because the joystick movement is not a sensitive enough measure or might point towards limited explanatory power of beta band activity for some patients. The current work has focused on the detection of a symptom-specific frequency within the beta frequency range for movement slowing. Similar principles could be translated to other spectral ranges and clinical signs such as tremor (Hirschmann et al., 2017, Shah et al., 2018), dystonia (Pina-Fuentes et al., 2019) or neuropsychiatric symptoms (Ricciardi et al., 2021). Indeed, our cohort comprised two tremor-dominant PD patients, who optimally should have been assessed using a tremor-specific assessment. Moreover, the present work focused on the fast oscillatory dynamics for beta burst-triggered aDBS, but frequency-specific dynamics may also be relevant for envelopes processed with longer smoothing time constants and proportional control algorithms (Arlotti et al., 2018, Bocci et al., 2021). Finally, our observations were made in a cohort with only recently implanted electrodes, hence the postoperative stun effect may have reduced the signal-to-noise ratio of LFP beta activity (Chen et al., 2006).

### 5.1 Outlook

In summary, this work emphasizes the potential of a data-driven selection of the feedback signal for optimizing aDBS, as opposed to simply rely on the PSD at rest. What is exemplarily shown here for movement velocity and PD can conceptually be translated to other clinical symptoms, DBS indications and electrophysiological biomarkers. Future work needs to be done to investigate and design an informative and user-friendly clinical-neurophysiological interrogation standard to select the optimal aDBS feedback signal. The recent introduction of neurostimulators with brain sensing capabilities will facilitate a systematic translation of both in-hospital and ambulatory neurophysiological assessment standards. This will not only leverage the clinical exploitation of aDBS but also enable comparability and interpretation of future aDBS clinical trials.

## Supplementary Material

Supplementary Materials

## Highlights

- The beta frequency strongest associated with motor slowing may differ from the individual beta peak at rest or largest movement-modulation frequency.
- The timing of beta bursts and triggering of adaptive deep brain stimulation differ across varying frequencies in the beta range.
- A clinical neurophysiological interrogation step can optimize the feedback signal selection for adaptive deep brain stimulation.

## Abbreviations

PD: Parkinson’s disease
LFP: local field potentials
STN: subthalamic nucleus
UPDRS: Unified Parkinson’s Disease Rating Scale
ERS: event-related synchronisation
ERD: event-related desynchronization
DBS: deep brain stimulation
AU: arbitrary unit
OVL: overlap
SEM: standard error of the mean
SD: standard deviation
PSD: power spectral density
IPG: implantable pulse generator

## References

[R1] Arlotti M, Marceglia S, Foffani G, Volkmann J, Lozano AM, Moro E (2018). Eight-hours adaptive deep brain stimulation in patients with Parkinson disease. Neurology.

[R2] Bocci T, Prenassi M, Arlotti M, Cogiamanian FM, Borrellini L, Moro E (2021). Eight-hours conventional versus adaptive deep brain stimulation of the subthalamic nucleus in Parkinson’s disease. NPJ Parkinsons Dis.

[R3] Brown P, Oliviero A, Mazzone P, Insola A, Tonali P, Di Lazzaro V (2001). Dopamine dependency of oscillations between subthalamic nucleus and pallidum in Parkinson’s disease. J Neurosci.

[R4] Chen CC, Pogosyan A, Zrinzo LU, Tisch S, Limousin P, Ashkan K (2006). Intra-operative recordings of local field potentials can help localize the subthalamic nucleus in Parkinson’s disease surgery. Exp Neurol.

[R5] Currà A, Berardelli A, Agostino R, Modugno N, Puorger CC, Accornero N (1997). Performance of sequential arm movements with and without advance knowledge of motor pathways in Parkinson’s disease. Mov Disord.

[R6] Devos D, Szurhaj W, Reyns N, Labyt E, Houdayer E, Bourriez JL (2006). Predominance of the contralateral movement-related activity in the subthalamo-cortical loop. Clin Neurophysiol.

[R7] di Biase L, Tinkhauser G, Martin Moraud E, Caminiti ML, Pecoraro PM, Di Lazzaro V (2021). Adaptive, personalized closed-loop therapy for Parkinson’s disease: biochemical, neurophysiological, and wearable sensing systems. Expert Rev Neurother.

[R8] Ding H, Groppa S, Muthuraman M (2022). Toward future adaptive deep brain stimulation for Parkinson’s disease: the novel biomarker - narrowband gamma oscillation. Neural Regen Res.

[R9] Doyle LM, Kühn AA, Hariz M, Kupsch A, Schneider GH, Brown P (2005). Levodopa-induced modulation of subthalamic beta oscillations during self-paced movements in patients with Parkinson’s disease. Eur J Neurosci.

[R10] Duchet B, Ghezzi F, Weerasinghe G, Tinkhauser G, Kühn AA, Brown P (2021). Average beta burst duration profiles provide a signature of dynamical changes between the ON and OFF medication states in Parkinson’s disease. PLoS Comput Biol.

[R11] Ernst MD (2004). Permutation Methods: A Basis for Exact Inference. Statist Sci.

[R12] Feldmann LK, Lofredi R, Neumann WJ, Al-Fatly B, Roediger J, Bahners BH (2022). Toward therapeutic electrophysiology: beta-band suppression as a biomarker in chronic local field potential recordings. NPJ Parkinsons Dis.

[R13] Fogelson N, Williams D, Tijssen M, van Bruggen G, Speelman H, Brown P (2006). Different functional loops between cerebral cortex and the subthalmic area in Parkinson’s disease. Cereb Cortex.

[R14] Gilron R, Little S, Perrone R, Wilt R, de Hemptinne C, Yaroshinsky MS (2021a). Long-term wireless streaming of neural recordings for circuit discovery and adaptive stimulation in individuals with Parkinson’s disease. Nat Biotechnol.

[R15] Gilron R, Little S, Wilt R, Perrone R, Anso J, Starr PA (2021b). Sleep-Aware Adaptive Deep Brain Stimulation Control: Chronic Use at Home With Dual Independent Linear Discriminate Detectors. Front Neurosci.

[R16] He S, Baig F, Mostofi A, Pogosyan A, Debarros J, Green AL (2021). Closed-Loop Deep Brain Stimulation for Essential Tremor Based on Thalamic Local Field Potentials. Mov Disord.

[R17] He S, Mostofi A, Syed E, Torrecillos F, Tinkhauser G, Fischer P (2020). Subthalamic beta-targeted neurofeedback speeds up movement initiation but increases tremor in Parkinsonian patients. Elife.

[R18] Herz DM, Little S, Pedrosa DJ, Tinkhauser G, Cheeran B, Foltynie T (2018). Mechanisms Underlying Decision-Making as Revealed by Deep-Brain Stimulation in Patients with Parkinson’s Disease. Curr Biol.

[R19] Hirschmann J, Schoffelen JM, Schnitzler A, van Gerven MAJ (2017). Parkinsonian rest tremor can be detected accurately based on neuronal oscillations recorded from the subthalamic nucleus. Clin Neurophysiol.

[R20] Iskhakova L, Rappel P, Deffains M, Fonar G, Marmor O, Paz R (2021). Modulation of dopamine tone induces frequency shifts in cortico-basal ganglia beta oscillations. Nat Commun.

[R21] Khawaldeh S, Tinkhauser G, Torrecillos F, He S, Foltynie T, Limousin P (2022). Balance between competing spectral states in subthalamic nucleus is linked to motor impairment in Parkinson’s disease. Brain.

[R22] Kuhn AA, Kupsch A, Schneider GH, Brown P (2006). Reduction in subthalamic 8–35 Hz oscillatory activity correlates with clinical improvement in Parkinson’s disease. Eur J Neurosci.

[R23] Little S, Beudel M, Zrinzo L, Foltynie T, Limousin P, Hariz M (2016a). Bilateral adaptive deep brain stimulation is effective in Parkinson’s disease. J Neurol Neurosurg Psychiatry.

[R24] Little S, Pogosyan A, Neal S, Zavala B, Zrinzo L, Hariz M (2013). Adaptive deep brain stimulation in advanced Parkinson disease. Ann Neurol.

[R25] Little S, Tripoliti E, Beudel M, Pogosyan A, Cagnan H, Herz D (2016b). Adaptive deep brain stimulation for Parkinson’s disease demonstrates reduced speech side effects compared to conventional stimulation in the acute setting. J Neurol Neurosurg Psychiatry.

[R26] Lofredi R, Neumann WJ, Bock A, Horn A, Huebl J, Siegert S (2018). Dopamine-dependent scaling of subthalamic gamma bursts with movement velocity in patients with Parkinson’s disease. Elife.

[R27] Lofredi R, Okudzhava L, Irmen F, Brücke C, Huebl J, Krauss JK (2022). Subthalamic beta bursts correlate with dopamine-dependent motor symptoms in 106 Parkinson’s patients. bioRxiv.

[R28] Lofredi R, Tan H, Neumann WJ, Yeh CH, Schneider GH, Kuhn AA (2019). Beta bursts during continuous movements accompany the velocity decrement in Parkinson’s disease patients. Neurobiol Dis.

[R29] Maris E, Oostenveld R (2007). Nonparametric statistical testing of EEG- and MEG-data. J Neurosci Methods.

[R30] Meidahl AC, Tinkhauser G, Herz DM, Cagnan H, Debarros J, Brown P (2017). Adaptive Deep Brain Stimulation for Movement Disorders: The Long Road to Clinical Therapy. Mov Disord.

[R31] Merk T, Peterson V, Köhler R, Haufe S, Richardson RM, Neumann WJ (2022). Machine learning based brain signal decoding for intelligent adaptive deep brain stimulation. Exp Neurol.

[R32] Muthuraman M, Bange M, Koirala N, Ciolac D, Pintea B, Glaser M (2020). Cross-frequency coupling between gamma oscillations and deep brain stimulation frequency in Parkinson’s disease. Brain.

[R33] Neumann WJ, Degen K, Schneider GH, Brucke C, Huebl J, Brown P (2016). Subthalamic synchronized oscillatory activity correlates with motor impairment in patients with Parkinson’s disease. Mov Disord.

[R34] Oswal A, Beudel M, Zrinzo L, Limousin P, Hariz M, Foltynie T (2016). Deep brain stimulation modulates synchrony within spatially and spectrally distinct resting state networks in Parkinson’s disease. Brain.

[R35] Oswal A, Cao C, Yeh CH, Neumann WJ, Gratwicke J, Akram H (2021). Neural signatures of hyperdirect pathway activity in Parkinson’s disease. Nat Commun.

[R36] Petrucci MN, Neuville RS, Afzal MF, Velisar A, Anidi CM, Anderson RW (2020). Neural closed-loop deep brain stimulation for freezing of gait. Brain Stimul.

[R37] Pina-Fuentes D, Little S, Oterdoom M, Neal S, Pogosyan A, Tijssen MAJ (2017). Adaptive DBS in a Parkinson’s patient with chronically implanted DBS: A proof of principle. Mov Disord.

[R38] Pina-Fuentes D, van Zijl JC, van Dijk JMC, Little S, Tinkhauser G, Oterdoom DLM (2019). The characteristics of pallidal low-frequency and beta bursts could help implementing adaptive brain stimulation in the parkinsonian and dystonic internal globus pallidus. Neurobiol Dis.

[R39] Priori A, Foffani G, Pesenti A, Tamma F, Bianchi AM, Pellegrini M (2004). Rhythm-specific pharmacological modulation of subthalamic activity in Parkinson’s disease. Exp Neurol.

[R40] Ricciardi L, Fischer P, Mostofi A, Tinkhauser G, Torrecillos F, Baig F (2021). Neurophysiological Correlates of Trait Impulsivity in Parkinson’s Disease. Mov Disord.

[R41] Rosa M, Arlotti M, Marceglia S, Cogiamanian F, Ardolino G, Fonzo AD (2017). Adaptive deep brain stimulation controls levodopa-induced side effects in Parkinsonian patients. Mov Disord.

[R42] Shah A, Nguyen TK, Peterman K, Khawaldeh S, Debove I, Shah SA (2023). Combining Multimodal Biomarkers to Guide Deep Brain Stimulation Programming in Parkinson Disease. Neuromodulation.

[R43] Shah SA, Tinkhauser G, Chen CC, Little S, Brown P (2018). Parkinsonian Tremor Detection from Subthalamic Nucleus Local Field Potentials for Closed-Loop Deep Brain Stimulation.

[R44] Sure M, Vesper J, Schnitzler A, Florin E (2021). Dopaminergic Modulation of Spectral and Spatial Characteristics of Parkinsonian Subthalamic Nucleus Beta Bursts. Front Neurosci.

[R45] Swann NC, de Hemptinne C, Thompson MC, Miocinovic S, Miller AM, Gilron R (2018). Adaptive deep brain stimulation for Parkinson’s disease using motor cortex sensing. J Neural Eng.

[R46] Tinkhauser G (2022). The present and future role of clinical neurophysiology for Deep Brain Stimulation. Clin Neurophysiol.

[R47] Tinkhauser G, Moraud EM (2021). Controlling Clinical States Governed by Different Temporal Dynamics With Closed-Loop Deep Brain Stimulation: A Principled Framework. Front Neurosci.

[R48] Tinkhauser G, Pogosyan A, Debove I, Nowacki A, Shah SA, Seidel K (2018a). Directional local field potentials: A tool to optimize deep brain stimulation. Mov Disord.

[R49] Tinkhauser G, Pogosyan A, Little S, Beudel M, Herz DM, Tan H (2017a). The modulatory effect of adaptive deep brain stimulation on beta bursts in Parkinson’s disease. Brain.

[R50] Tinkhauser G, Pogosyan A, Tan H, Herz DM, Kuhn AA, Brown P (2017b). Beta burst dynamics in Parkinson’s disease OFF and ON dopaminergic medication. Brain.

[R51] Tinkhauser G, Shah SA, Fischer P, Peterman K, Debove I, Nygyuen K (2019). Electrophysiological differences between upper and lower limb movements in the human subthalamic nucleus. Clin Neurophysiol.

[R52] Tinkhauser G, Torrecillos F, Duclos Y, Tan H, Pogosyan A, Fischer P (2018b). Beta burst coupling across the motor circuit in Parkinson’s disease. Neurobiol Dis.

[R53] Tinkhauser G, Torrecillos F, Pogosyan A, Mostofi A, Bange M, Fischer P (2020). The Cumulative Effect of Transient Synchrony States on Motor Performance in Parkinson’s Disease. J Neurosci.

[R54] Torrecillos F, Tinkhauser G, Fischer P, Green AL, Aziz TZ, Foltynie T (2018). Modulation of Beta Bursts in the Subthalamic Nucleus Predicts Motor Performance. J Neurosci.

[R55] van Rheede JJ, Feldmann LK, Busch JL, Fleming JE, Mathiopoulou V, Denison T (2022). Diurnal modulation of subthalamic beta oscillatory power in Parkinson’s disease patients during deep brain stimulation. NPJ Parkinsons Dis.

[R56] Velisar A, Syrkin-Nikolau J, Blumenfeld Z, Trager MH, Afzal MF, Prabhakar V (2019). Dual threshold neural closed loop deep brain stimulation in Parkinson disease patients. Brain Stimul.

[R57] Wiest C, Tinkhauser G, Pogosyan A, Bange M, Muthuraman M, Groppa S (2020). Local field potential activity dynamics in response to deep brain stimulation of the subthalamic nucleus in Parkinson’s disease. Neurobiol Dis.

[R58] Wiest C, Tinkhauser G, Pogosyan A, He S, Baig F, Morgante F (2021). Subthalamic deep brain stimulation induces finely-tuned gamma oscillations in the absence of levodopa. Neurobiol Dis.

[R59] Yeh CH, Al-Fatly B, Kühn AA, Meidahl AC, Tinkhauser G, Tan H (2020). Waveform changes with the evolution of beta bursts in the human subthalamic nucleus. Clin Neurophysiol.

