# Supplementary material for: Clinical neurophysiological interrogation of motor slowing: A critical step towards tuning adaptive deep brain stimulation
Source: Clin Neurophysiol. Author manuscript; Available in PMC 2024 May 8. (PMC7615935; doi:10.1016/j.clinph.2023.04.013)
Supplement: Supplementary Materials [file EMS195569-supplement-Supplementary_Materials.docx]

**Supplementary material**

**Clinical neurophysiological interrogation of motor slowing: A critical step towards tuning adaptive deep brain stimulation**

**Methods**:

**Subjects and surgery**

| Subj | Gender,  M/F | Age,  y | Disease Duration,  y | Pre-Op  UPDRS-III,  OFF | Pre-Op  UPDRS-III,  ON | Predominant symptoms | Time  Extern, d | DBS lead | Contact pair | Hand | Site |
| --- | --- | --- | --- | --- | --- | --- | --- | --- | --- | --- | --- |
| 1 | M | 61 | 16 | 50 | 30 | Akinetic-rigid | 3 | Bost | L12 | R | St. George London |
| 2 | M | 59 | 6 | 48 | 14 | Akinetic-rigid | 5 | Medt | L23 | R | St. George London |
| 3 | M | 65 | 15 | 77 | 27 | Akinetic-rigid, tremor | 5 | Bost | L01 | R | St. George London |
| 4 | M | 48 | 17 | 71 | 37 | Tremor | 3 | Bost | R12 | R | St. George London |
| 5 | M | 54 | 7 | 38 | 24 | Tremor | 5 | Bost | R12 | R | St. George London |
| 6 | M | 56 | 16 | 51 | 19 | Akinetic-rigid, tremor | 4 | Medt | L12 | R | St. George London |
| 7 | M | 66 | 15 | 57 | 34 | Akinetic-rigid, tremor | 4 | Medt | L12 | R | St. George London |
| 8 | F | 66 | 10 | 53 | 30 | Akinetic-rigid | 4 | Bost | L01 | R | St. George London |
| 9 | M | 61 | 10 | 31 | 19 | Akinetic-rigid, tremor | 3 | Medt | L01 | R | Mainz, University Hospital |
| 10 | F | 67 | 13 | 18 | 15 | Akinetic-rigid, tremor | 3 | Medt | L23 | R | Mainz, University Hospital |
| 11 | M | 77 | 7 | 35 | 29 | Akin-rigid | 3 | Medt | L23 | R | Mainz, University Hospital |
| 12 | M | 65 | 10 | 37 | 9 | Akinetic-rigid, tremor | 6 | Medt | L23 | R | Kings College London |
| 13 | F | 70 | 20 | 54 | 19 | Akinetic-rigid, tremor | 6 | Medt | L01 | R | Kings College London |
| 14 | M | 69 | 17 | 37 | 18.5 | Akinetic-rigid, tremor | 6 | Medt | L23 | R | Kings College London |
| 15 | M | 68 | 12 | 40 | 17 | Akinetic-rigid, tremor | 6 | Medt | L12 | R | Kings College London |
| **Mean ± SEM (*n*)** | **M(12); F(3)** | **63.4±1.9** | **12.7±1.1** | **46.5±3.9** | **22.8±2.1** |  | **Median 4 (3-6)** |  | **18-8 ±1.1** |  |  |

Supplementary Table 1:

**Clinical details.** Subj=subject; M=male; F=female; y=years; d=days; R=right; L=left; Pre-Op=pre-operative; UPDRS=Unified Parkinson’s disease rating scale Part III; Bost=Boston Scientific; Medt=Medtronic; SEM=standard error of the mean. This table has been previously published (Tinkhauser *et al.*, 2020).

**Experimental set-up and behavioural task**

The following methodological description has been detailed in the original publication (Tinkhauser *et al.*, 2020), in summary: Assessments were performed after withdrawal of the dopaminergic medication. LFPs were recorded using the TMSi-Porti amplifier (TMS International, Netherlands) with signals low-pass filtered at 550 Hz, sampled at 2048 Hz and common average referenced. A bipolar contact arrangement between the four electrode levels (directional contacts of one level were joined together to form one ‘contact’) was set offline and each DBS lead afforded three bipolar signals for the left (L01, L12, L23) and right (R01, R12, R23) STN. This bipolar montage also served to limit the effects of volume conduction from distant sources (Marmor *et al.*, 2017). Using the same system, the timing of cue-presentation, the displacement of the joystick and the signal from an accelerometer taped to the hand used to control the joystick were co-registered. One bipolar channel contralateral to the active hand was selected and used for further analyses (Supplementary Table 1). To primarily pick up the activity from the motor region of the STN, we selected the channel with the highest resting beta activity, or, in the case of similar levels of beta power across channels, the one with the strongest beta modulation during voluntary hand movements (Tinkhauser *et al.*, 2018a; Tinkhauser *et al.*, 2019; Shah *et al.*, 2022). The task was developed in-house: Subjects sat comfortably in front of a computer monitor and held a joystick placed on the table. The position of the joystick was displayed as a red circle and localised at the bottom centre of the screen when in resting position. At the top of the screen, distributed on a half-circle, three equally spaced, grey circle targets were shown (left side, middle, right side). Subjects were instructed to make a rapid movement from the resting position in the direction and through the target when one of the three grey targets changed colour to green (GO-cue). The quick nature of the response was stressed. Moreover, to minimize any corrective movements, we did not provide visual feedback of the cursor position during the ongoing movement. The position of the red cursor was presented at rest, disappeared after movement onset, and reappeared once the movement trajectory went beyond the target. The inter-trial interval was 7 seconds plus up to 2.5 seconds burst detection period. After initial familiarization (10-20 trials) of the task, we aimed to obtain a minimum number of 60 trials, subdivided in several blocks.


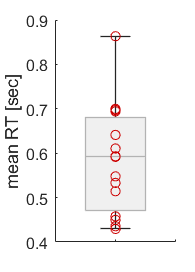


Supplementary Figure 1:

**Reaction time.** Shows the distribution of the mean reaction times across trials for each subject. The reaction time was measured from the go-cue to movement onset. The median mean reaction time across subjects was 0.59 seconds. RT=reaction time.

**Offline behavioural analysis**

For this study, we re-used the processed behavioural data from the original study (Tinkhauser *et al.*, 2020). The description of the processing steps are detailed in the original publication and are again summarized here: after visual data inspection using Spike2 Software (CED Cambridge Electronic design limited, United Kingdom), trials contaminated by artefacts, by movements during the resting period or failed trials (e.g. subject did not move) were removed from the dataset. All further analyses were performed using custom-written MATLAB scripts (version R2020b; MathWorks). Motor performance was characterized by the peak velocity (PV) of the joystick movement. This parameter has been selected because of the well described association between bradykinesia and basal ganglia beta bursts (Tinkhauser *et al.*, 2017a; Tinkhauser *et al.*, 2017b; Torrecillos *et al.*, 2018; Lofredi *et al.*, 2019). For this, the position of the red joystick cursor was differentiated to derive the displacement of the joystick over time (movement velocity). Movement onset was defined as the time when the joystick velocity exceeded five-times the standard deviation of the signal at rest. PV was then defined as the maximum velocity in the direction of the target. We only considered trials with a reaction time (measured from GO cue to movement onset) less than 1.5 seconds, and thereafter also rejected trials in which PV or reaction time exceeded 2.5 times the SD from the mean. After these data processing steps an average number of 82 trials [range 33 to 149] were available for further analyses.

**Results**:

Supplementary Figure 2:


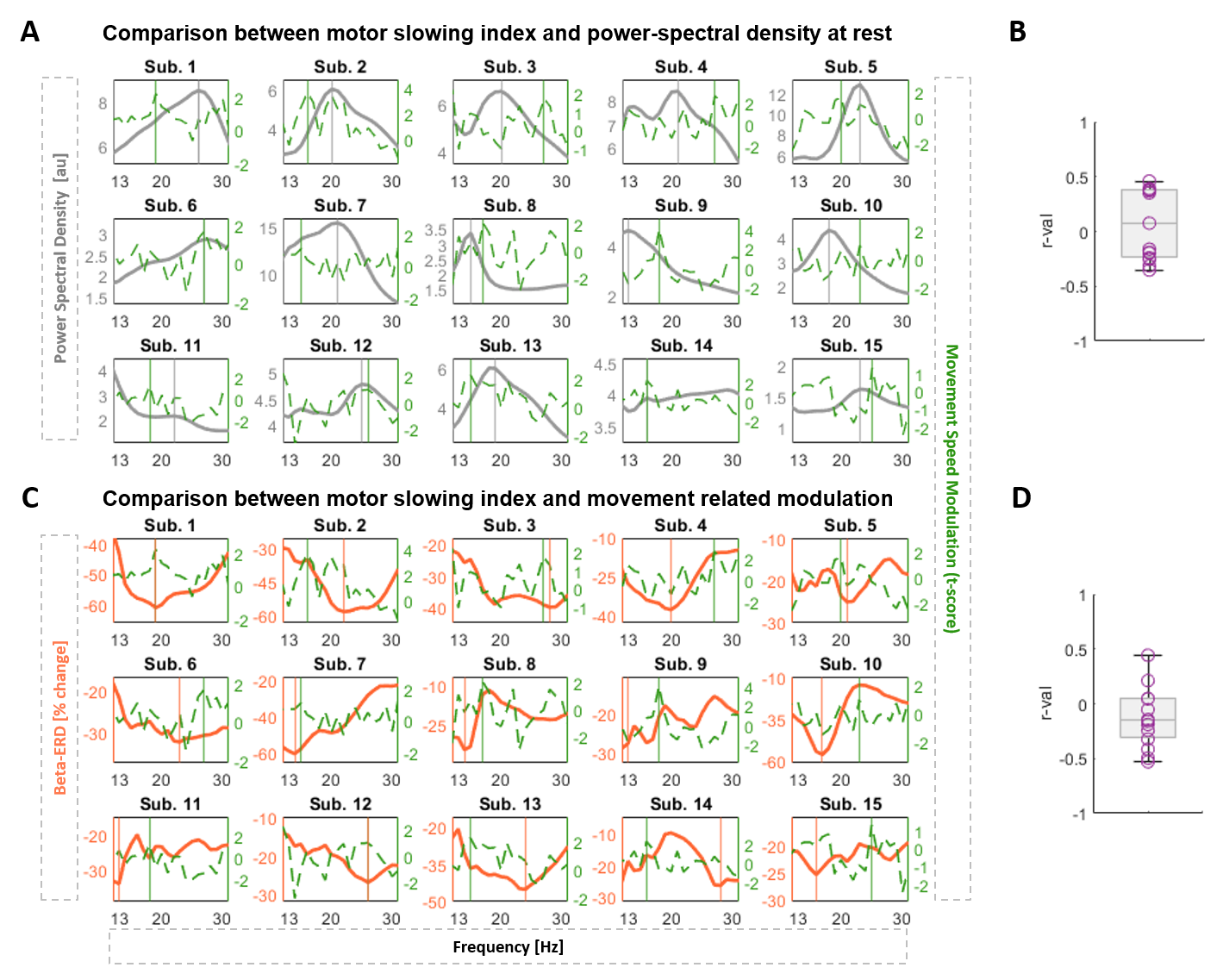


**Clinical and spectral profile across subjects. A)** Illustrates for every subject (n=15) and beta frequency the PSD of the LFPs recorded at rest (grey line) and the burst duration dependent motor modulation represented as the t-score (dashed green line). A high t-score indicates a stronger association between burst duration and motor slowing. In some instances (e.g. sub. 2), both the PSD and t-score curves show a similar magnitude across the beta range, while in others (e.g. sub. 3) even an inverse relationship can be seen. The vertical lines mark the individual beta peak (grey line) and the individual MSF i.e. frequency strongest associated with the motor slowing (green line). **B)** Shows the distribution of the r-values derived from the Spearman’s correlation between the individual PSD curve and t-score curves across subjects. The median r-value is 0.08, ranging from -0.35 to 0.46. **C)** Illustrates for every subject and beta frequency the averaged movement-related beta desynchronization, the % beta ERD (orange line), and the burst duration dependent motor modulation represented as t-score (dashed green line, same as above). The vertical lines mark the individual frequency with the strongest beta ERD (orange line) and the individual MSF (green line). **D)** Shows the distribution of the r-values derived for each subject from the Spearman’s correlation between the individual beta ERD curve and t-score curves. The median r-value is 0.14, ranging from -0.53 to 0.44. Note for B) and D): If the curves would consistently co-fluctuate, i.e. if the peaks and troughs would approximately co-occur, we should only see positive r-values that are close to 1 for B, and only negative r-values, closer to -1 for D (because in the orange line the trough denotes the beta frequency that would be selected based on movement-related ERD). The fact that the r-values are both positive and negative, ranging a broad span, summarizes that the peaks (and ERD troughs), and thus the selected aDBS frequency, can be very different. Note, bursts are defined using the 75^th^ percentile amplitude threshold. Sub.=Subjects; AU=arbitrary unit; MSF=motor slowing frequency; LFP=local field potentials; PSD=power spectral density; ERD=event-related-desynchronization..


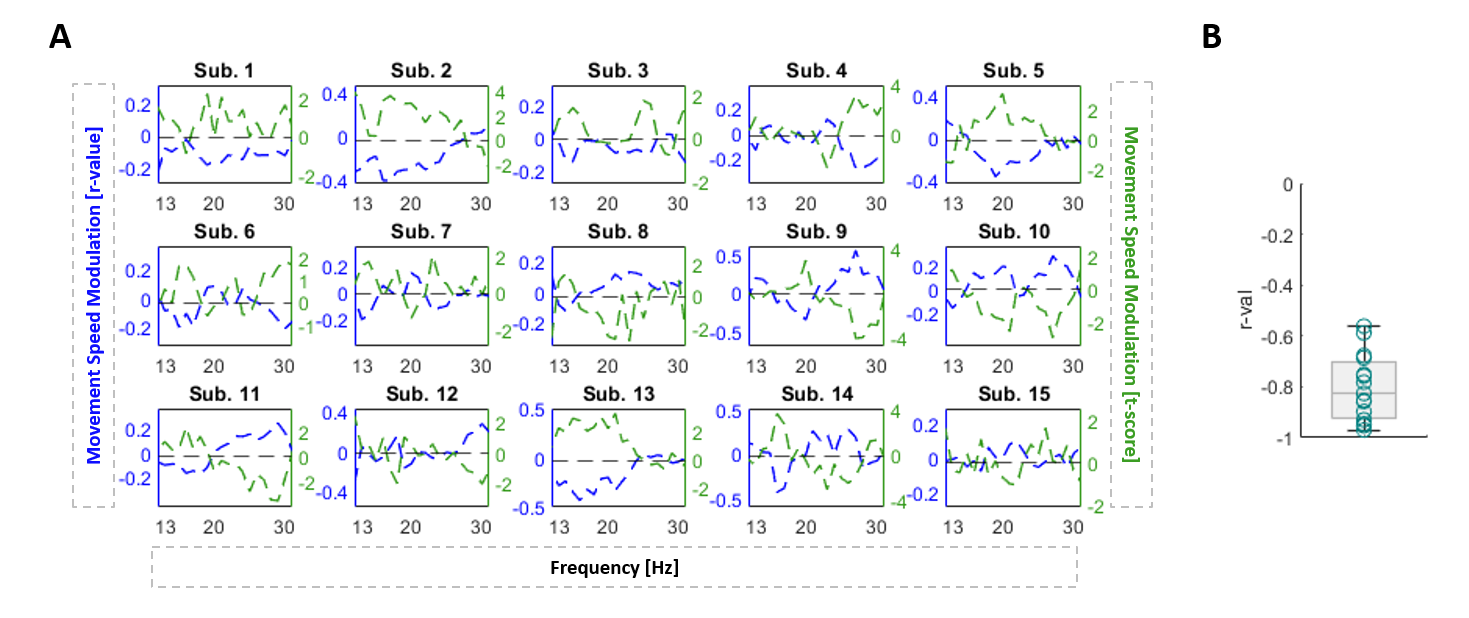


Supplementary Figure 3:

**Spectro-Behavioural Relationship represented as t-score and r-value. A)** Illustrates for every subject (n=15) and beta frequency the burst amplitude dependent motor modulation represented as t-score and the r-value. The dashed green line presents the t-score derived by an independent two-sample t-test comparing the movement peak velocity of trials median split based on their pre-movement mean burst amplitude. A high t-score indicates a stronger association between burst amplitude and motor slowing. The dashed blue line in comparison presents the r-value derived by a Spearman correlation between the movement peak velocity and the pre-movement mean burst amplitude across trials. The two curves show a strong inverse correlation. **B)** Shows the distribution of the r-values derived from the Spearman’s correlation between the t-score curve and the r-value curve across subjects. The median r-value is -0.83, ranging from -0.97 to -0.56.

| 2.5s | 1 |  |  |
| --- | --- | --- | --- |
| 2s | 0.72 ± 0.15 | 1 |  |
| 1.5s | 0.60 ± 0.20 | 0.71 ± 0.16 | 1 |
|  | 2.5s | 2s | 1.5s |

Supplementary Table 2:

**Impact of burst detection period prior movement onset.** The beta burst related motor slowing across the beta frequency range (expressed as t-score curves) was calculated for different burst time windows starting from the following timings prior to movement onset: -2.5s, -2.0s, -1.5s, while the -2.5s represent our default. This matrix illustrates the Pearson’s correlation coefficients resulting from the correlation between the t-score curves for the different burst detection time windows within subject and then averaged across the group. This shows that the magnitudes and distribution of the t-score curves are relatively comparable across different burst time windows.


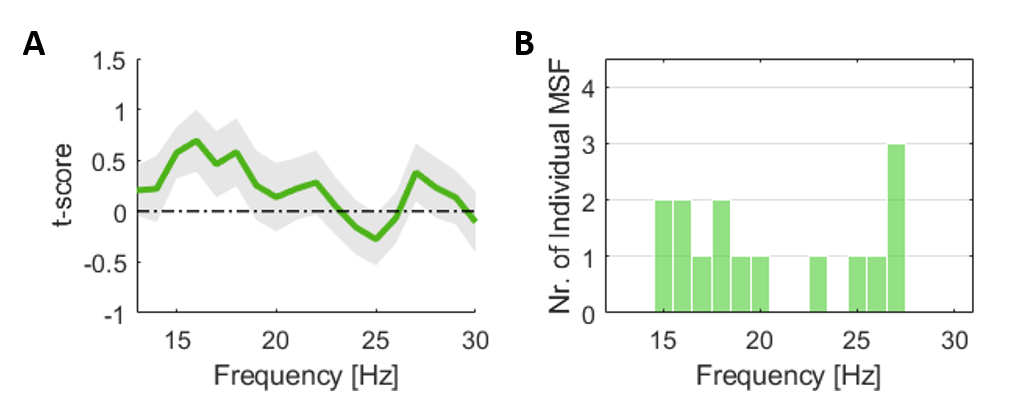


Supplementary Figure 4:

**Impact of burst duration on motor slowing across beta frequencies. A)** Shows the t-scores as indicator of motor slowing across the beta frequency range and averaged across subjects (n=15). Values are represented as mean ± SEM. No significant spectral associations with motor slowing at group level have been found. **B)** Illustrates the distribution of the individual MSFs, i.e. the individual beta frequency strongest associated with motor slowing. In 9 out of 15 subjects the individual MSF is located in the low beta range (13 to 20 Hz) and in 6 out of 15 cases in the high beta range (21 to 30 Hz). MSF=motor slowing frequency; SEM=standard error of the mean.


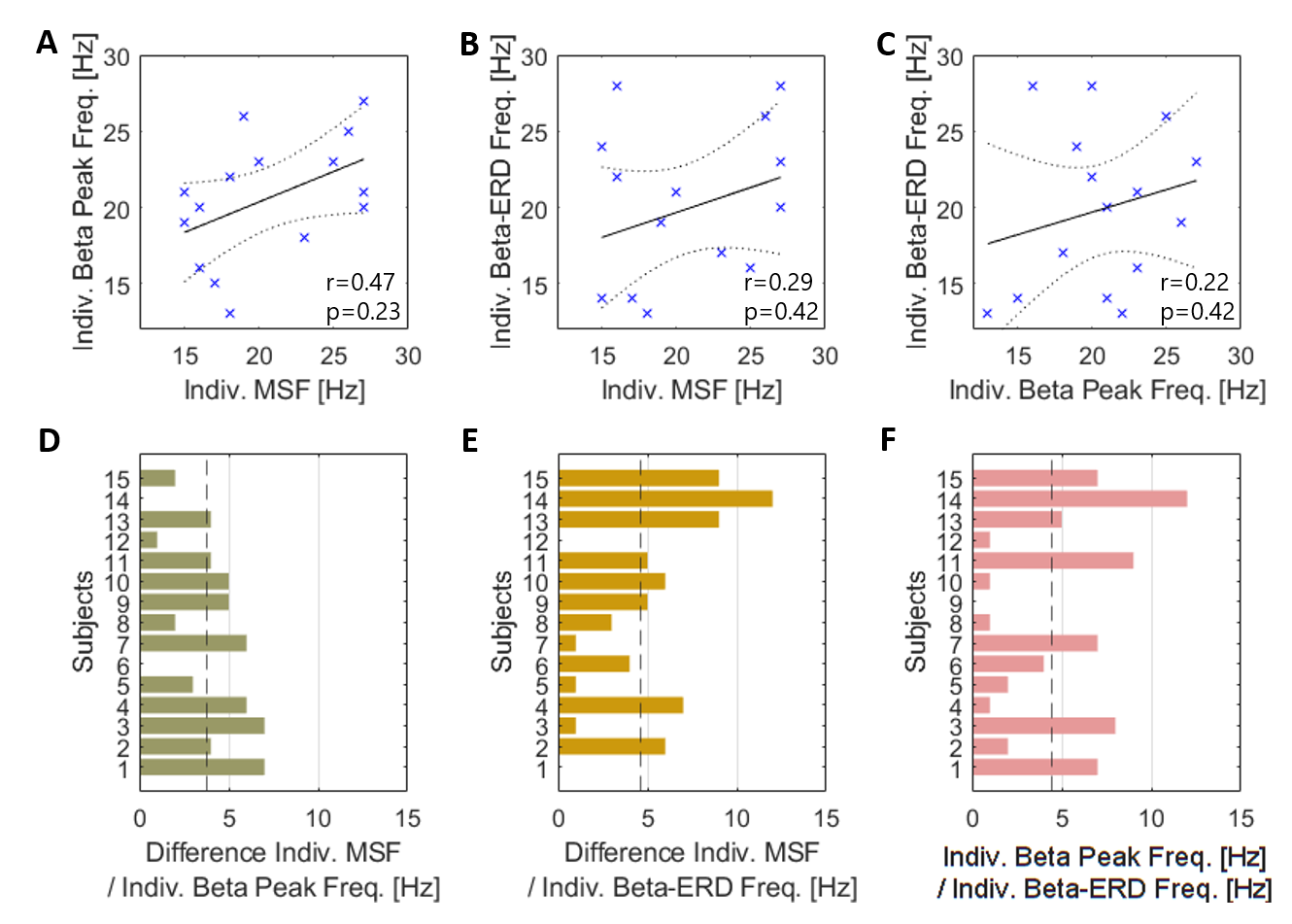


Supplementary Figure 5:

**Spectral relationship between individual MSF, beta peak and beta ERD frequencies. A)** Spearman’s correlation between the individual MSFs and the individual beta peak frequencies (r=0.47, p=0.23). **B)** Spearman’s correlation between the individual MSF and the frequencies with strongest beta ERD (r=0.29, p=0.42). **C)** Spearman’s correlation between the individual beta peak frequencies and the frequencies with strongest beta ERD. (r=0.22, p=0.42). **D)** Illustrates for every subject the absolute difference between the individual MSF and the individual beta peak frequency, which on average corresponds to 3.7 Hz (dashed line). **E)** Shows the absolute difference between the individual MSF and the individual beta ERD frequency, which is 4.6 Hz on average. **F)** Shows the absolute difference between the individual beta peak frequency and the individual beta ERD frequency, that on average corresponds to 4.5 Hz. MSF=motor slowing frequency; ERD=event-related-desynchronization; SEM=standard error of the mean.

| **Friedman’s ANOVA Table** | | | | | | | | | | | | | |
| --- | --- | --- | --- | --- | --- | --- | --- | --- | --- | --- | --- | --- | --- |
| Source | | Sum of Squares | | df | | Mean Squares | | | Chi-square | | p-value for Chi-square | | |
| Columns | | 308897.9 | | 7 | | 44128.3 | | | 255.29 | | 2.07442e-51 | | |
| Interactions | | 552.4 | | 14 | | 39.5 | | |  | |  | | |
| Error | | 122519.2 | | 336 | | 364.6 | | |  | |  | | |
| Total | | 431969.5 | | 359 | |  | | |  | |  | | |
| **Post-hoc analysis** | | | | | | | | | | | | | |
| Groups | p(1) | | p(2) | | p(3) | | p(4) | p(5) | | p(6) | | p(7) | p(8) |
| MSF – BP | 0.7148 | | 0.7148 | | 0.7148 | | 0.7148 | 0.7148 | | 0.7148 | | 0.7148 | 0.7148 |
| MSF – BE | 0.7826 | | 0.7826 | | 0.7826 | | 0.7826 | 0.7826 | | 0.7826 | | 0.7826 | 0.7869 |
| BP – BE | 0.2301 | | 0.2301 | | 0.2301 | | 0.2301 | 0.2301 | | 0.2301 | | 0.8077 | 0.4086 |

Supplementary Table 3:

**Statistical comparison of Percentage Burst Overlapping, main figure 7B:** Upper panel: Friedman’s test to evaluate the decrease in %OVL when deviating from the individual beta candidate frequencies. Lower panel: pairwise post-hoc analysis using Wilcoxon signed rank tests between beta candidate frequencies for the different frequency groups 1 to 8 Hz. Only FDR corrected p-values are displayed. df=degrees of freedom; Chi-square=Friedman’s chi-square statistic; MSF= individual motor slowing frequency; BP= individual beta peak frequency; BE=individual beta ERD frequency; p=p-value; ERD=event-related-desynchronization.


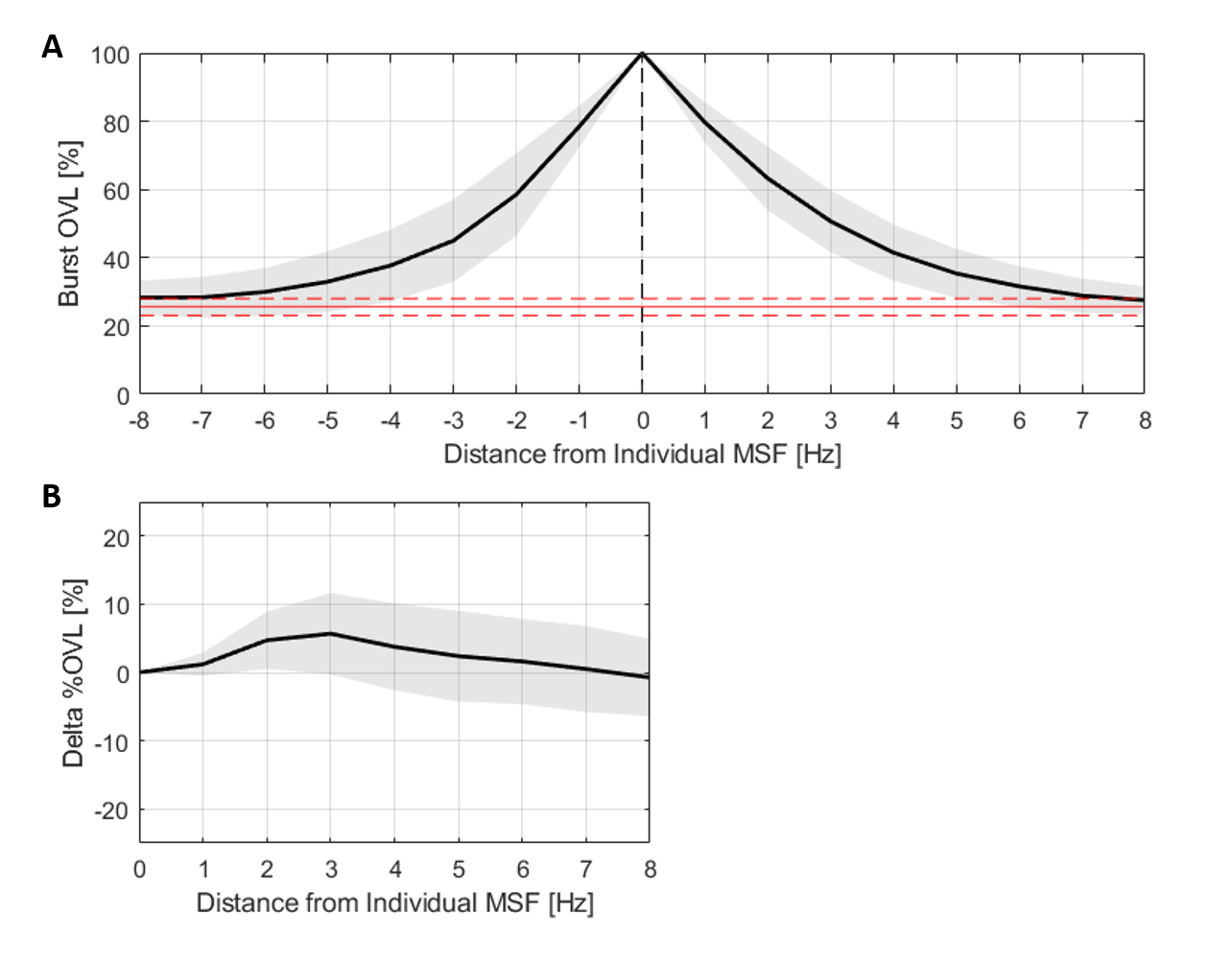


Supplementary Figure 6:

**Percentage Burst Overlapping Relative to Individual MSF.** **A.** The slope illustrates the frequency-bin wise % change in mean burst OVL with incremental deviation from the individual MSF in both directions. The red horizontal lines mark the percentage burst overlapping (%OVL) by chance ± 1 SD (25.53% +2.47). The %OVL decreases to 79.65% with +1 Hz, to 78.46% with -1 Hz, to 50.70% with +3 Hz and to 45.02% with -3 Hz deviation from the individual MSF. Values are represented as mean ± SD. **B.** Illustrates the difference in the frequency-bin wise % change in mean burst OVL with incremental deviation from the individual MSF between the left and the right side of the individual MSF. Values are represented as mean ± SD. Overall, A and B show that deviations in both directions lead to a similar decay in %OVL. Bursts are defined using the 75th percentile amplitude threshold. MSF=motor slowing frequency; OVL=overlapping; SD=standard deviation.


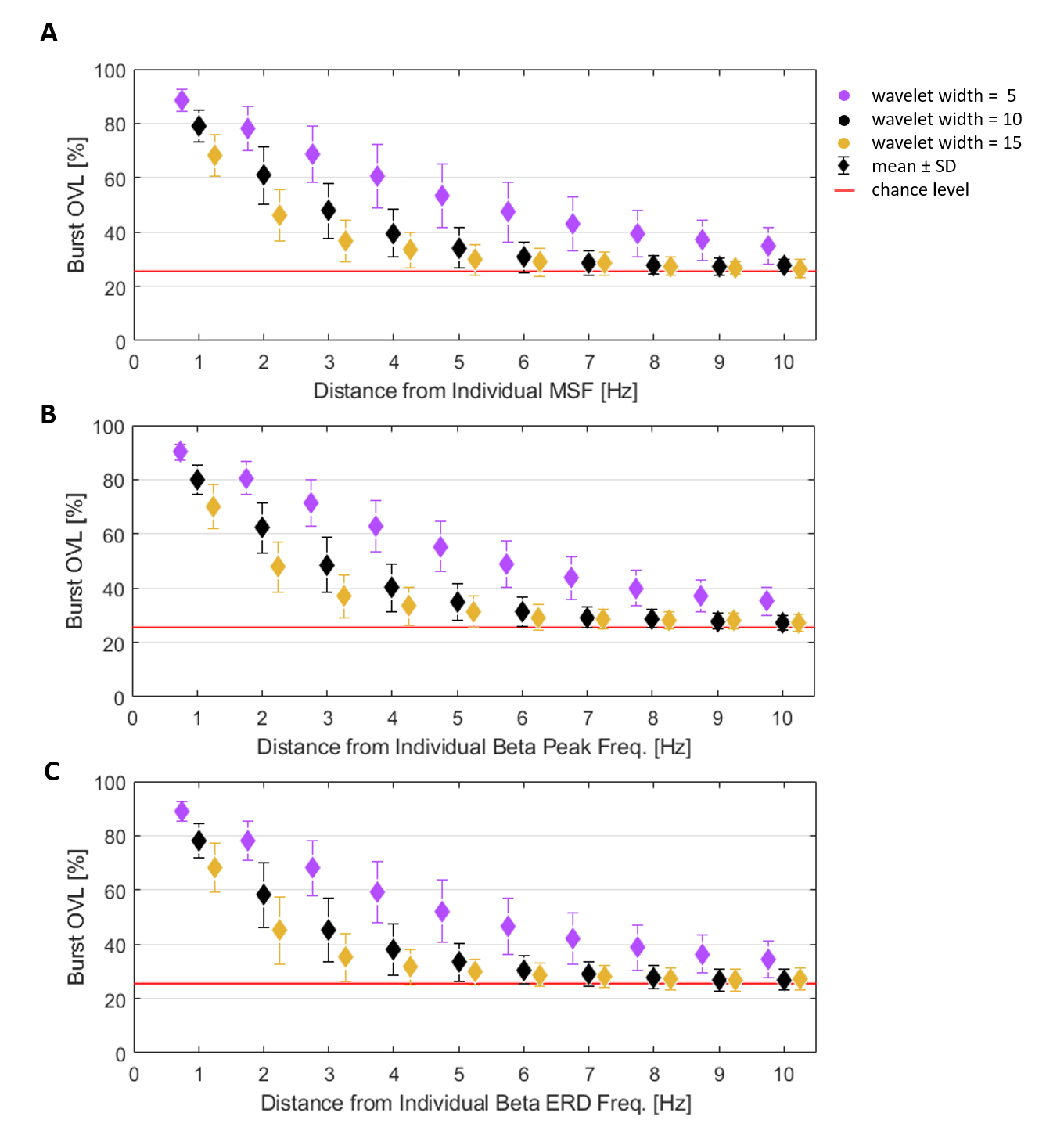


Supplementary Figure 7:

**Impact of chosen wavelet width on burst overlapping.** Illustrates the frequency-bin wise change in mean %OVL (± SD) with incremental deviation from the three individual beta frequencies (A: individual MSF, B: individual beta peak, C: individual beta ERD) when using three different wavelet width (wt 5, wt 10, wt 15) for the LFP rest recording decomposition. Note, the % change derived from the deviations to both the left and right side of the individual MSF have been averaged. The %OVL by chance of the three wavelet width is depicted by the horizontal red lines (averaged: 25.71, wt 5: 26.25, wt 10: 25.53, wt 15: 25.36). For all three individual beta frequencies, a steeper %OVL slope is seen when using a higher frequency resolution and a flattening of the %OVL slope when using a lower frequency resolution. Values are represented as mean ± SD. Bursts are defined using the 75th percentile amplitude threshold. MSF=motor slowing frequency; ERD=event-related-desynchronization; LFP=local field potential; OVL=overlap; SD=standard deviation; wt=wavelet width.


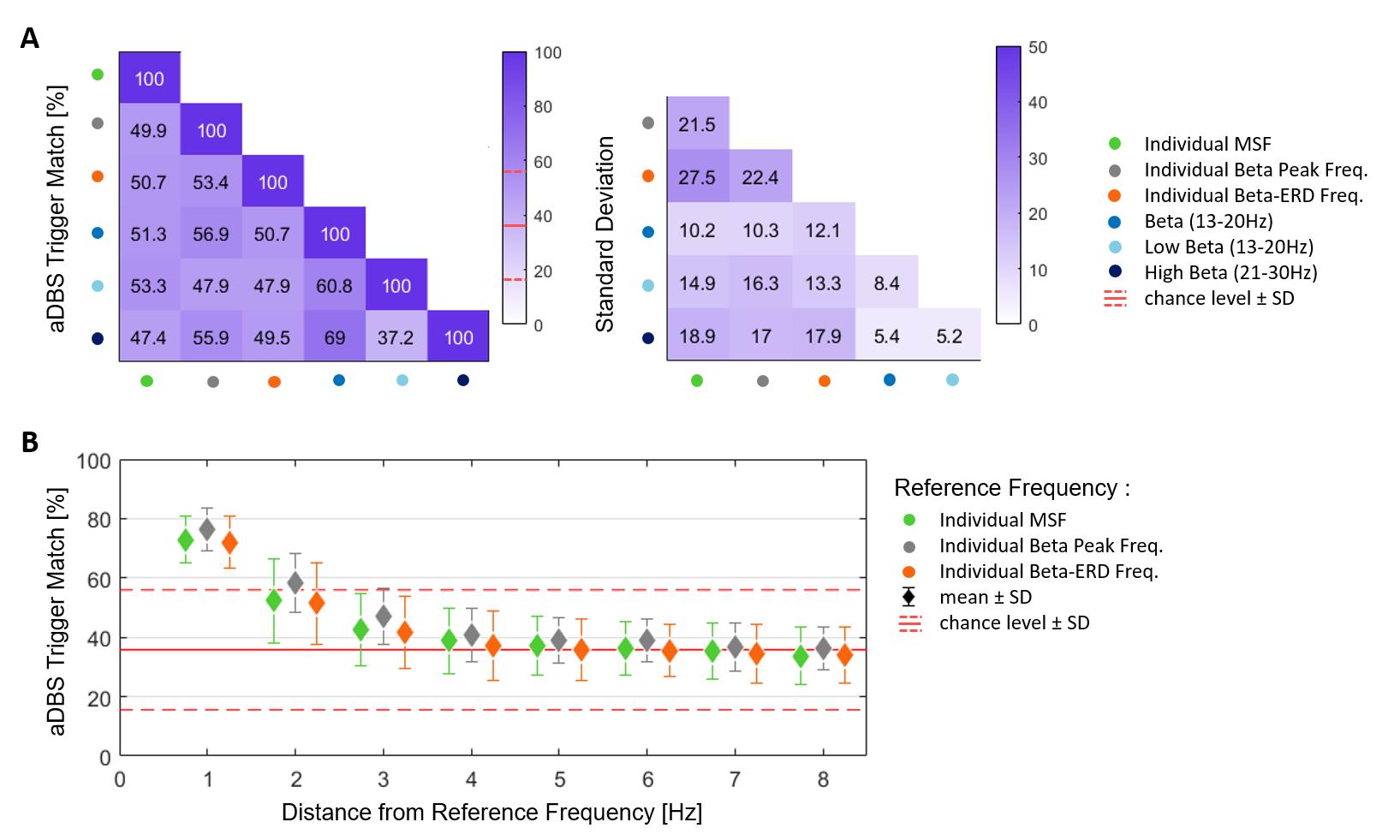


Supplementary Figure 8:

**Adaptive DBS Trigger Match, 50^th^ percentile threshold.** The matrix (left) illustrates the average % aDBS trigger match between the different beta candidate frequencies (MSF, beta peak, beta ERD, entire beta (13-30 Hz), low beta (13-20 Hz) and high beta band (21-30 Hz)), averaged across subjects (n=15). The %trigger-match is defined by how often the burst onset (stimulation trigger) of one candidate frequency coincides with the stimulation trigger tolerance area of the comparison frequency (and vice versa). The chance level ± 1 SD corresponded to 35.80% ± 20.18%. The %trigger-match ranges from 37.2% (low beta – high beta) to 69% (entire beta – high beta). The matrix (right) illustrates the corresponding standard deviation values. **B)** Illustrates the frequency-bin wise change in mean % aDBS trigger match (± SD) with incremental deviation from the individual reference frequencies: the individual MSF (green), the individual beta peak frequency (grey), and the individual beta ERD frequency (orange). Note, the % changes derived from the deviations to both the left and right side have been averaged. The horizontal red lines depict the %trigger match by chance ± 1 SD. The %trigger-match decreases to 72.81% with 1 Hz and to 42.74% with 3 Hz deviation from the individual MSF, to 76.25% with 1 Hz and to 46.97% with 3 Hz deviation from the individual beta peak frequency and to 72.07% with 1 Hz and to 41.68% with 3 Hz deviation from the individual beta ERD frequency. Values are represented as mean ± SD. Bursts are defined using the 50^th^ percentile amplitude threshold. MSF=motor slowing frequency; ERD=event-related-desynchronization; SD=standard deviation.

| **Friedman’s ANOVA Table** | | | | | | | | | | | | |  |
| --- | --- | --- | --- | --- | --- | --- | --- | --- | --- | --- | --- | --- | --- |
| Source | Sum of Squares | | df | | Mean Squares | | | Chi-square | | p-value for Chi-square | | |  |
| Columns | 255651.6 | | 7 | | 36521.7 | | | 211.29 | | 4.6548e-42 | | |  |
| Interactions | 1851.3 | | 14 | | 132.2 | | |  | |  | | |  |
| Error | 174457.1 | | 336 | | 364.6 | | |  | |  | | |  |
| Total | 431960 | | 359 | |  | | |  | |  | | |  |
| **Post-hoc analysis** | | | | | | | | | | | | | |
| Groups | p(1) | p(2) | | p(3) | | p(4) | p(5) | | p(6) | | p(7) | p(8) | |
| MSF – BP | 0. 4631 | 0. 4631 | | 0. 4631 | | 0. 4631 | 0. 4631 | | 0. 4631 | | 0. 4631 | 0. 4631 | |
| MSF – BE | 0. 9460 | 0. 9460 | | 0. 9460 | | 0. 9460 | 0. 9460 | | 0. 9460 | | 0. 9460 | 0. 9460 | |
| BP – BE | 0. 3465 | 0. 3465 | | 0. 3465 | | 0. 3465 | 0. 3465 | | 0. 5293 | | 0. 5416 | 0. 4767 | |

Supplementary Table 4:

**Statistical comparison of Percentage adaptive DBS Trigger Match, main figure 8B:** Upper panel: Friedman’s test to evaluate the decrease in %trigger match when deviating from the individual beta candidate frequencies. Lower panel: pairwise post-hoc analysis using Wilcoxon signed rank tests between beta candidate frequencies for the different frequency groups 1 to 8 Hz. Only FDR corrected p-values are displayed. df=degrees of freedom; Chi-square=Friedman’s chi-square statistic; MSF= individual motor slowing frequency; BP= individual beta peak frequency; BE=individual beta ERD frequency; p=p-value; ERD=event-related-desynchronization.

| **Friedman’s ANOVA Table** | | | | | | | | | | | |
| --- | --- | --- | --- | --- | --- | --- | --- | --- | --- | --- | --- |
| Source | Sum of Squares | | df | | Mean Squares | | Chi-square | | p-value for Chi-square | | |
| Columns | 207376.9 | | 6 | | 34562.8 | | 223.6 | | 1.77985e-45 | | |
| Interactions | 148.8 | | 12 | | 12.4 | |  | |  | | |
| Error | 81842.8 | | 294 | | 278.4 | |  | |  | | |
| Total | 289368.5 | | 314 | |  | |  | |  | | |
| **Post-hoc analysis** | | | | | | | | | | | |
| Groups | p(1) | p(2) | | p(3) | | p(4) | | p(5) | | p(6) | p(7) |
| MSF – BP | 0.1578 | 0.1937 | | 0.1578 | | 0.1578 | | 0.1578 | | 0.1578 | 0.1578 |
| MSF – BE | 0.9780 | 0.9780 | | 0.9780 | | 0.9780 | | 0.9780 | | 0.9780 | 0.9780 |
| BP – BE | 0.0236* | 0.0676 | | 0.0232* | | 0.0184* | | 0.0184* | | 0.0184* | 0.0162* |

Supplementary Table 5:

**Statistical comparison of Percentage adaptive DBS Trigger Match, main figure 8C:** Upper panel: Friedman’s test to evaluate the decrease in %trigger match when combining the target frequency with an incremental number of neighbouring frequency bins. Lower panel: pairwise post-hoc analysis using Wilcoxon signed rank tests between beta candidate frequencies for the different frequency groups 1 to 7 Hz. Only FDR corrected p-values are displayed. df=degrees of freedom; Chi-square=Friedman’s chi-square statistic; MSF= individual motor slowing frequency; BP= individual beta peak frequency; BE=individual beta ERD frequency; p=p-value; ERD=event-related-desynchronization.
